# Supplementary material for: Maternal Socioeconomic Status and the Initiation and Duration of Breastfeeding in Western Europe Versus Southern Africa: A Systematic Review—A Contribution from the ConcePTION Project
Source: Nutrients. 2025 Mar 8;17(6):946. doi: 10.3390/nu17060946 (PMC11944666; doi:10.3390/nu17060946)
Supplement: Supplementary file 1 [file nutrients-17-00946-s001.zip › nutrients-3496793-supplementary.pdf]

File S1. Search Strategy

PubMed (MEDLINE)

(“Lesotho”[Mesh] OR "Lesotho"[tiab] OR “Maseru”[tiab] OR "Basutoland"[tiab] OR “Botswana”[Mesh] OR "Botswana"[tiab] OR “Gaborone”[tiab] OR "Bechuanaland"[tiab] OR "Kalahari"[tiab] OR “Namibia”[Mesh] OR "Namibia"[tiab] OR “Zambezi”[tiab] OR “Kavango”[tiab] OR “Kunene”[tiab] OR “Omusati”[tiab] OR “Ohangwena”[tiab] OR “Oshana”[tiab] OR “Oshikoto”[tiab] OR “Omaheke”[tiab] OR “Otjozondjupa”[tiab] OR “Erongo”[tiab] OR “Khomas”[tiab] OR “Hardap”[tiab] OR “Karas”[tiab] OR “Windhoek”[tiab] OR “Eswatini”[Mesh] OR "Eswatini"[tiab] OR “Mbabane”[tiab] OR “Lobamba”[tiab] OR "Swaziland"[tiab] OR "Hhohho”[tiab] OR “Lubombo”[tiab] OR “Manzini”[tiab] OR “Shiselweni”[tiab] OR “South Africa”[Mesh] OR "South Africa"[tiab] OR “Cape Town”[tiab] OR “Eastern Cape”[tiab] OR “Free State”[tiab] OR “Gauteng”[tiab] OR “KwaZulu-Natal”[tiab] OR “Limpopo”[tiab] OR “Mpumalanga”[tiab] OR “Northern Cape”[tiab] OR “Western Cape”[tiab] OR "Belgium"[Mesh] OR "Belgium"[tiab] OR “Flemish”[tiab] OR “Walloon” [tiab] OR “Brussels”[tiab] OR “Flanders”[tiab] OR “Wallonia”[tiab] OR “Dutch”[tiab] OR "Netherlands"[Mesh] OR “Netherlands”[tiab] OR "Holland"[tiab] OR “Amsterdam”[tiab] OR "Luxembourg"[Mesh] OR "Luxemb*"[tiab] OR “benelux”[tiab] OR "Germany"[Mesh] OR "German*"[tiab] OR “Berlin”[tiab] OR "France"[Mesh] OR "France"[tiab] OR “Auvergne-Rhône-Alpes”[tiab] OR “Burgundy-Franche-Comté”[tiab] OR “Brittany”[tiab] OR “Center-Val de Loire”[tiab] OR “Great East”[tiab] OR “Normandy”[tiab] OR “New Aquitaine”[tiab] OR “Occitanie”[tiab] OR “Pays de la Loire”[tiab] OR “Provence-Alpes-Côte d'Azur”[tiab] OR “Paris”[tiab] OR “French”[tiab] OR "Monaco"[Mesh] OR "Monaco"[tiab] OR "Austria"[Mesh] OR "Austria"[tiab] OR “Vienna”[tiab] OR "Switzerland"[Mesh] OR "Switzerland"[tiab] OR “Bern”[tiab] OR "Liechtenstein"[Mesh] OR "Liechtenstein"[tiab] OR “Leichtenstein”[tiab]) AND ("initiat*"[tiab] OR "duration"[tiab] OR "extent"[tiab] OR "period"[tiab] OR "commitment"[tiab] OR "contin*"[tiab] OR “Start*”[tiab] OR “begin*”[tiab] OR “induct*”[tiab] OR “endur*”[tiab] OR “persistence”[tiab] OR “prolong*”[tiab] OR “sust*”[tiab] OR “preserv*”[tiab] OR “maintaining”[tiab] OR “engage*”[tiab]) AND ("Breast Feeding"[Mesh] OR "breast fe*"[tiab] OR "breastfe*"[tiab]

OR "milk sharing"[tiab] OR "wet nursing"[tiab] OR "Milk, Human"[Mesh] OR "human milk"[tiab] OR "breast milk"[tiab] OR "maternal milk"[tiab] OR "mother milk"[tiab] OR "woman milk"[tiab] OR "breast pumping*"[tiab] OR “lactation”[Mesh] OR “lactation”[tiab] OR “Infant Feeding”[tiab] OR “Maternal Feeding”[tiab] OR “Child Feeding”[tiab] OR “Mother’s milk”[tiab]) AND ("Socioeconomic factors"[Mesh] OR "Factor*"[ti] OR "Determinant*"[ti] OR "Aspect*"[ti] OR "Characteristic*"[ti] OR "socioeconomic*"[tiab] OR "socio economic*”[tiab] OR “Living standard*”[tiab] OR “Social*”[tiab] OR “Economic*”[tiab] OR "population*"[tiab] OR "income"[tiab] OR "disparit*"[tiab] OR "ineq*"[tiab] OR "Social Status”[Mesh] OR "caste*”[tiab] OR “vulnerab*”[tiab] OR “Vulnerability”[Mesh] OR “Education*”[tiab] OR “Literacy”[tiab] OR “housing instability”[tiab] OR “poverty”[tiab] OR “employment”[tiab] OR “Rank”[tiab] OR “achievement”[tiab] OR “indig*”[tiab] OR “Ghetto*”[tiab] OR “Slum*”[tiab] OR “poorness”[tiab] OR “occupation”[tiab] OR “profession”[tiab] OR “job”[tiab] OR “jobs”[tiab] OR “realization*”[tiab] OR “Success”[tiab] OR “qualification”[tiab] OR “Sociodemographic factors”[Mesh] OR “Sociodemographic*”[tiab] OR “Socio demographic*“[tiab] OR “Cross-cultural comparison”[Mesh] OR “cultural”[tiab] OR “ethnic”[tiab] OR “racial”[tiab] OR “minorit*”[tiab] OR “Ethnic and racial minorities”[Mesh] OR “Minority Groups”[Mesh] OR “Cultural deprivation”[Mesh] OR “Cultural diversity”[Mesh] OR “Cultural characteristics”[Mesh] OR “diversit*”[tiab] OR “Hierarchy, social”[Mesh] OR “Social Marginalization”[Mesh] OR “Living condition*”[tiab] OR “Wealth”[tiab] OR “Classism”[tiab] OR ”Illiteracy”[tiab] OR “Unemployment”[tiab])

Embase (Elsevier)

('lesotho'/exp OR 'lesotho':ti,ab,kw OR 'maseru':ti,ab,kw OR 'basutoland':ti,ab,kw OR 'botswana'/exp OR 'botswana':ti,ab,kw OR 'gaborone':ti,ab,kw OR 'bechuanaland':ti,ab,kw OR 'kalahari':ti,ab,kw OR 'namibia'/exp OR 'namibia':ti,ab,kw OR 'zambezi':ti,ab,kw OR 'kavango':ti,ab,kw OR 'kunene':ti,ab,kw OR 'omusati':ti,ab,kw OR 'ohangwena':ti,ab,kw OR 'oshana':ti,ab,kw OR 'oshikoto':ti,ab,kw OR 'omaheke':ti,ab,kw OR 'otjozondjupa':ti,ab,kw OR 'erongo':ti,ab,kw OR 'khomas':ti,ab,kw OR 'hardap':ti,ab,kw OR 'karas':ti,ab,kw OR 'windhoek':ti,ab,kw OR 'eswatini'/exp OR 'eswatini':ti,ab,kw OR 'mbabane':ti,ab,kw OR 'lobamba':ti,ab,kw OR 'swaziland':ti,ab,kw OR 'hhohho':ti,ab,kw OR 'lubombo':ti,ab,kw OR 'manzini':ti,ab,kw OR 'shiselweni':ti,ab,kw OR 'south africa'/exp OR 'south africa':ti,ab,kw OR

'cape town':ti,ab,kw OR 'eastern cape':ti,ab,kw OR 'free state':ti,ab,kw OR 'gauteng':ti,ab,kw OR 'kwazulu-natal':ti,ab,kw OR 'limpopo':ti,ab,kw OR 'mpumalanga':ti,ab,kw OR 'northern cape':ti,ab,kw OR 'western cape':ti,ab,kw OR 'belgium'/exp OR 'belgium':ti,ab,kw OR 'brussels capital region'/exp OR 'flemish':ti,ab,kw OR 'walloon':ti,ab,kw OR 'brussels':ti,ab,kw OR 'flanders'/exp OR 'flanders':ti,ab,kw OR 'wallonia'/exp OR 'wallonia':ti,ab,kw OR 'dutch':ti,ab,kw OR 'netherlands'/exp OR 'netherlands':ti,ab,kw OR 'holland':ti,ab,kw OR 'amsterdam':ti,ab,kw OR 'luxembourg'/exp OR 'luxemb*':ti,ab,kw OR 'benelux'/exp OR 'benelux':ti,ab,kw OR 'germany'/exp OR 'german*':ti,ab,kw OR 'berlin':ti,ab,kw OR 'france'/exp OR 'france':ti,ab,kw OR 'auvergne-rhône-alpes':ti,ab,kw OR 'burgundy-franche-comté':ti,ab,kw OR 'brittany':ti,ab,kw OR 'center-val de loire':ti,ab,kw OR 'great east':ti,ab,kw OR 'normandy':ti,ab,kw OR 'new aquitaine':ti,ab,kw OR 'occitanie':ti,ab,kw OR 'pays de la loire':ti,ab,kw OR 'provence-alpes-côte d azur':ti,ab,kw OR 'paris':ti,ab,kw OR ‘french’:ti,ab,kw OR 'monaco'/exp OR 'monaco':ti,ab,kw OR 'liechtenstein'/exp OR 'liechtenstein':ti,ab,kw OR 'leichtenstein':ti,ab,kw OR 'austria'/exp OR 'austria':ti,ab,kw OR ‘vienna’:ti,ab,kw OR 'switzerland'/exp OR 'switzerland':ti,ab,kw OR ’bern’:ti,ab,kw) AND ('initiat*':ti,ab,kw OR 'duration':ti,ab,kw OR 'extent':ti,ab,kw OR 'period':ti,ab,kw OR 'commitment':ti,ab,kw OR 'contin*':ti,ab,kw OR 'start*':ti,ab,kw OR 'begin*':ti,ab,kw OR 'induct*':ti,ab,kw OR 'endur*':ti,ab,kw OR 'persistence':ti,ab,kw OR 'prolong*':ti,ab,kw OR 'sust*':ti,ab,kw OR 'preserv*':ti,ab,kw OR 'maintaining':ti,ab,kw OR 'engage*':ti,ab,kw) AND ('breast feeding'/exp OR 'breast fe*':ti,ab,kw OR 'breastfe*':ti,ab,kw OR 'milk sharing':ti,ab,kw OR 'wet nursing':ti,ab,kw OR 'breast milk expression'/exp OR 'breast milk':ti,ab,kw OR 'human milk':ti,ab,kw OR 'maternal milk':ti,ab,kw OR 'mother milk':ti,ab,kw OR 'woman milk':ti,ab,kw OR 'breast pumping*':ti,ab,kw OR 'lactation'/exp OR 'lactation':ti,ab,kw OR 'infant feeding':ti,ab,kw OR 'maternal feeding':ti,ab,kw OR 'child feeding':ti,ab,kw OR 'mother s milk':ti,ab,kw) AND ('socioeconomics'/exp OR 'health disparity'/exp OR 'social status'/exp OR 'social determinants of health'/exp OR 'sociodemographics'/exp OR 'factor*':ti OR 'determinant*':ti OR 'aspect*':ti OR 'characteristic*':ti OR 'socioeconomic*':ti,ab,kw OR 'socio-economic*':ti,ab,kw OR 'social*':ti,ab,kw OR 'economic*':ti,ab,kw OR 'living standard*':ti,ab,kw OR 'population*':ti,ab,kw OR 'income':ti,ab,kw OR 'disparit*':ti,ab,kw OR 'ineq*':ti,ab,kw OR 'caste*':ti,ab,kw OR 'vulnerab*':ti,ab,kw OR 'education*':ti,ab,kw OR 'literacy':ti,ab,kw OR 'housing instability':ti,ab,kw OR 'poverty':ti,ab,kw OR 'employment':ti,ab,kw OR 'rank':ti,ab,kw OR 'achievement':ti,ab,kw OR 'indig*':ti,ab,kw OR 'ghetto*':ti,ab,kw OR 'slum*':ti,ab,kw OR 'poorness':ti,ab,kw OR 'occupation':ti,ab,kw OR

'profession':ti,ab,kw OR 'job':ti,ab,kw OR 'jobs':ti,ab,kw OR 'realisation*':ti,ab,kw OR 'succes':ti,ab,kw OR 'qualification':ti,ab,kw OR 'sociodemographic*':ti,ab,kw OR 'socio-demographic*':ti,ab,kw OR 'cultural':ti,ab,kw OR 'ethnic':ti,ab,kw OR 'racial':ti,ab,kw OR 'minorit*':ti,ab,kw OR 'diversit*':ti,ab,kw OR “Living condition*”:ti,ab,kw OR “Wealth”:ti,ab,kw OR “Classism”:ti,ab,kw OR ”Illiteracy”:ti,ab,kw OR “Unemployment”:ti,ab,kw)

CINAHL (EBSCO)

(MH "Lesotho") OR TI (“Lesotho” OR “Maseru” OR "Basutoland") OR AB (“Lesotho” OR “Maseru” OR "Basutoland") OR (MH "Botswana") OR TI (“Botswana” OR “Gaborone” OR "Bechuanaland" OR "Kalahari") OR AB (“Botswana” OR “Gaborone” OR "Bechuanaland" OR "Kalahari") OR (MH “Namibia”) OR TI (“Namibia" OR “Zambezi” OR “Kavango” OR “Kunene” OR “Omusati” OR “Ohangwena” OR “Oshana” OR “Oshikoto” OR “Omaheke” OR “Otjozondjupa” OR “Erongo” OR “Khomas” OR “Hardap” OR “Karas” OR “Windhoek”) OR AB (“Namibia" OR “Zambezi” OR “Kavango” OR “Kunene” OR “Omusati” OR “Ohangwena” OR “Oshana” OR “Oshikoto” OR “Omaheke” OR “Otjozondjupa” OR “Erongo” OR “Khomas” OR “Hardap” OR “Karas” OR “Windhoek”) OR TI (“Eswatini’ OR “Mbabane” OR “Lobamba” OR "Swaziland" OR "Hhohho” OR “Lubombo” OR “Manzini” OR “Shiselweni”) OR AB (“Eswatini’ OR “Mbabane” OR “Lobamba” OR "Swaziland" OR "Hhohho” OR “Lubombo” OR “Manzini” OR “Shiselweni”) OR (MH "South Africa") OR TI (“South Africa” OR “Cape Town” OR “Eastern Cape” OR “Free State” OR “Gauteng” OR “KwaZulu-Natal” OR “Limpopo” OR “Mpumalanga” OR “Northern Cape” OR “Western Cape”) OR AB (“South Africa” OR “Cape Town” OR “Eastern Cape” OR “Free State” OR “Gauteng” OR “KwaZulu-Natal” OR “Limpopo” OR “Mpumalanga” OR “Northern Cape” OR “Western Cape”) OR (MH "Belgium") OR TI ("Belgium" OR “Flemish” OR “Walloon” OR “Brussels” OR “Flanders” OR “Wallonia” OR “Dutch”) OR AB ("Belgium" OR “Flemish” OR “Walloon” OR “Brussels” OR “Flanders” OR “Wallonia” OR “Dutch”) OR (MH "Netherlands") OR TI (“Netherlands” OR "Holland" OR “Amsterdam”) OR AB (“Netherlands” OR "Holland" OR “Amsterdam”) OR (MH "Luxembourg") OR TI (“Luxembourg” OR "Luxemb*") OR AB (“Luxembourg” OR "Luxemb*") OR TI (“benelux”) OR AB (“Benelux”) OR (MH"Germany") OR TI (“Germany” OR "German*" OR “Berlin”) OR AB (“Germany” OR "German*" OR “Berlin”) OR (MH "France") OR TI ("France" OR “Auvergne-Rhône-

Alpes” OR “Burgundy-Franche-Comté” OR “Brittany” OR “Center-Val de Loire” OR “Great East” OR “Normandy” OR “New Aquitaine” OR “Occitanie” OR “Pays de la Loire” OR “Provence-Alpes-Côte d'Azur” OR “Paris” OR “French”) OR AB ("France" OR “Auvergne-Rhône-Alpes” OR “Burgundy-Franche-Comté” OR “Brittany” OR “Center-Val de Loire” OR “Great East” OR “Normandy” OR “New Aquitaine” OR “Occitanie” OR “Pays de la Loire” OR “Provence-Alpes-Côte d'Azur” OR “Paris” OR “French”) OR (MH "Monaco") OR TI ("Monaco") OR AB (“Monaco”) OR (MH "Austria") OR TI ("Austria" OR “Vienna”) OR AB ("Austria" OR “Vienna”) OR (MH "Switzerland") OR TI ("Switzerland" OR “Bern”) OR AB ("Switzerland" OR “Bern”) OR (MH "Liechtenstein") OR TI ("Liechtenstein" OR “Leichtenstein”) OR AB ("Liechtenstein" OR “Leichtenstein”) AND TI ("initiat*" OR "duration" OR "extent" OR "period" OR "contin*" OR “Start*” OR “begin*” OR “induct*” OR “endur*” OR “persistence” OR “prolong*” OR “sust*” OR “preserv*” OR “maintaining” OR “engage*”) OR AB ("initiat*" OR "duration" OR "extent" OR "period" OR "contin*" OR “Start*” OR “begin*” OR “induct*” OR “endur*” OR “persistence” OR “prolong*” OR “sust*” OR “preserv*” OR “maintaining” OR “engage*”) OR (MH “Commitment”) OR TI (“Commitment”) OR AB (“Commitment”) AND (MH "Breast Feeding") OR (MH ”Milk, Human) OR (MH “Lactation”) OR (MH “Infant Feeding”) OR TI ("breast fe*" OR "breastfe*" OR "milk sharing" OR "wet nursing" OR "human milk" OR "breast milk" OR "maternal milk" OR "mother milk" OR "woman milk" OR "breast pumping*" OR “lactation” OR “Infant Feeding” OR “Maternal Feeding” OR “Child Feeding” OR “Mother’s milk”) OR AB ("breast fe*" OR "breastfe*" OR "milk sharing" OR "wet nursing" OR "human milk" OR "breast milk" OR "maternal milk" OR "mother milk" OR "woman milk" OR "breast pumping*" OR “lactation” OR “Infant Feeding” OR “Maternal Feeding” OR “Child Feeding” OR “Mother’s milk”) AND (MH "Socioeconomic Factors") OR (MH "Socioeconomic Disparities in Health") OR (MH “Population”) OR (MH “Social Status”) OR (MH “Income”) OR (MH “Social determinants of health”) OR (MH “Health Inequities”) OR (MH “Social Class”) OR (MH “Vulnerability”) OR (MH “Poverty+”) OR (MH “Employment”) OR (MH "Achievement+") OR (MH "Sociodemographic Factors") OR (MH "Minority Groups") OR (MH "Cultural Diversity+") OR (MH “Low and middle income countries”) OR (MH “Unemployment”) OR (MH “Economic factors”) OR (MH “economic status”) OR (MH “educational status”) OR (MH “Employment”) OR (MH “illiteracy”) OR (MH “income”) OR (MH “literacy”) OR (MH “Low Socioeconomic Status”) OR (MH “Occupations and Professions”) OR (MH “Social factors”) OR (MH “Ethnic Groups”) OR TI ("Factor*" OR "Determinant*" OR "Aspect*" OR

"Characteristic*" OR "socioeconomic*" OR "socio economic*” OR “Living standard*” OR “Social*” OR “Economic*” OR "population*" OR "income" OR "disparit*" OR "ineq*" OR "caste*” OR “vulnerab*” OR “Education*” OR “Literacy” OR “housing instability” OR “poverty” OR “employment” OR “Rank” OR “achievement” OR “indig*” OR “Ghetto*” OR “Slum*” OR “poorness” OR “occupation” OR “profession” OR “job” OR “jobs” OR “realization*” OR “Success” OR “qualification” OR “Sociodemographic*” OR “Socio demographic*“ OR “cultural” OR “ethnic” OR “racial” OR “minorit*” OR “diversit*” OR “Living condition*” OR “Wealth” OR “Classism” OR ”Illiteracy” OR “Unemployment”) OR AB ("socioeconomic*" OR "socio economic*” OR “Living standard*” OR “Social*” OR “Economic*” OR "population*" OR "income" OR "disparit*" OR "ineq*" OR "caste*” OR “vulnerab*” OR “Education*” OR “Literacy” OR “housing instability” OR “poverty” OR “employment” OR “Rank” OR “achievement” OR “indig*” OR “Ghetto*” OR “Slum*” OR “poorness” OR “occupation” OR “profession” OR “job” OR “jobs” OR “realization*” OR “Success” OR “qualification” OR “Sociodemographic*” OR “Socio demographic*“ OR “cultural” OR “ethnic” OR “racial” OR “minorit*” OR “diversit*” OR “Living condition*” OR “Wealth” OR “Classism” OR ”Illiteracy” OR “Unemployment”)

Central (The Cochrane Library)

([mh “Lesotho”] OR ‘Lesotho’:ti,ab,kw OR ‘Maseru’:ti,ab,kw OR ‘Basutoland’:ti,ab,kw OR [mh “Botswana”] OR ’Botswana’:ti,ab,kw OR ‘Gaborone’:ti,ab,kw OR ‘Bechuanaland’:ti,ab,kw OR ‘Kalahari’:ti,ab,kw OR [mh “Namibia”] OR ’Namibia’:ti,ab,kw OR ‘Zambezi’:ti,ab,kw OR ‘Kavango’:ti,ab,kw OR ‘Kunene’:ti,ab,kw OR ‘Omusati’:ti,ab,kw OR ‘Ohangwena’:ti,ab,kw OR ‘Oshana’:ti,ab,kw OR ‘Oshikoto’:ti,ab,kw OR ‘Omaheke’:ti,ab,kw OR ‘Otjozondjupa’:ti,ab,kw OR ‘Erongo’:ti,ab,kw OR ‘Khomas’:ti,ab,kw OR ‘Hardap’:ti,ab,kw OR ‘Karas’:ti,ab,kw OR ‘Windhoek’:ti,ab,kw OR [mh “Eswatini”] OR ’Eswatini’:ti,ab,kw OR ‘Mbabane’:ti,ab,kw OR ‘Lobamba’:ti,ab,kw OR ‘Swaziland’:ti,ab,kw OR ‘Hhohho’:ti,ab,kw OR ‘Lubombo’:ti,ab,kw OR ‘Manzini’:ti,ab,kw OR ‘Shiselweni’:ti,ab,kw OR [mh “South Africa”] OR ’South Africa’:ti,ab,kw OR ‘Cape Town’:ti,ab,kw OR ‘Eastern Cape’:ti,ab,kw OR ‘Free State’:ti,ab,kw OR ‘Gauteng’:ti,ab,kw OR ‘KwaZulu-Natal’:ti,ab,kw OR ‘Limpopo’:ti,ab,kw OR ‘Mpumalanga’:ti,ab,kw OR ‘Northern Cape’:ti,ab,kw OR ‘Western Cape’:ti,ab,kw OR [mh “Belgium”] OR ‘Belgium’:ti,ab,kw OR ‘Flemish’:ti,ab,kw OR ‘Walloon’:ti,ab,kw OR ‘Brussels’:ti,ab,kw OR

‘Flanders’:ti,ab,kw OR ‘Wallonia’:ti,ab,kw OR ‘Dutch’:ti,ab,kw OR [mh “Netherlands”] OR ‘Netherlands’:ti,ab,kw OR ‘Holland’:ti,ab,kw OR ‘Amsterdam’:ti,ab,kw OR [mh “Luxembourg”] OR ‘Luxemb*’:ti,ab,kw OR ‘benelux’:ti,ab,kw OR [mh “Germany”] OR ‘German*’:ti,ab,kw OR ‘Berlin’:ti,ab,kw OR [mh “France”] OR ‘France’:ti,ab,kw OR ‘Auvergne-Rhône-Alpes’:ti,ab,kw OR ‘Burgundy-Franche-Comté’:ti,ab,kw OR ‘Brittany’:ti,ab,kw OR ‘Center-Val de Loire’:ti,ab,kw OR ‘Great East’:ti,ab,kw OR ‘Normandy’:ti,ab,kw OR ‘New Aquitaine’:ti,ab,kw OR ‘Occitanie’:ti,ab,kw OR ‘Pays de la Loire’:ti,ab,kw OR ‘Provence-Alpes-Côte d'Azur’:ti,ab,kw OR ‘Paris’:ti,ab,kw OR ‘French’:ti,ab,kw OR [mh “Monaco”] OR ‘Monaco’:ti,ab,kw OR [mh “Austria”] OR ‘Austria’:ti,ab,kw OR ‘Vienna’:ti,ab,kw OR [mh “Switzerland”] OR ‘Switzerland’:ti,ab,kw OR ‘Bern’:ti,ab,kw OR [mh “Liechtenstein”] OR ‘Liechtenstein’:ti,ab,kw OR ‘Leichtenstein’:ti,ab,kw) AND (‘initiat*’:ti,ab,kw OR ‘duration’:ti,ab,kw OR ‘extent’:ti,ab,kw OR ‘period’:ti,ab,kw OR ‘commitment’:ti,ab,kw OR ‘contin*’:ti,ab,kw OR ‘Start*’:ti,ab,kw OR ‘begin*’:ti,ab,kw OR ‘induct*’:ti,ab,kw OR ‘endur*’:ti,ab,kw OR ‘persistence’:ti,ab,kw OR ‘prolong*’:ti,ab,kw OR ‘sust*’:ti,ab,kw OR ‘preserv*’:ti,ab,kw OR ‘maintaining’:ti,ab,kw OR ‘engage*’:ti,ab,kw) AND ([mh “Breast Feeding”] OR ‘breast fe*’:ti,ab,kw OR ‘breastfe*’:ti,ab,kw OR ‘milk sharing’:ti,ab,kw OR ‘wet nursing’:ti,ab,kw OR [mh “Milk, Human”] OR ‘human milk’:ti,ab,kw OR ‘breast milk’:ti,ab,kw OR ‘maternal milk’:ti,ab,kw OR ‘mother milk’:ti,ab,kw OR ‘woman milk’:ti,ab,kw OR ‘breast pumping*’:ti,ab,kw OR [mh “lactation”] OR ‘lactation’:ti,ab,kw OR ‘Infant Feeding’:ti,ab,kw OR ‘Maternal Feeding’:ti,ab,kw OR ‘Child Feeding’:ti,ab,kw OR ‘Mother’s milk’:ti,ab,kw) AND ([mh “Socioeconomic factors”] OR ‘Factor*’:ti OR ‘Determinant*’:ti OR ‘Aspect*’:ti OR ‘Characteristic*’:ti OR ‘socioeconomic*’:ti,ab,kw OR ‘socio economic*’:ti,ab,kw OR ‘Living standard*’:ti,ab,kw OR ‘Social*’:ti,ab,kw OR ‘Economic*’:ti,ab,kw OR ‘population*’:ti,ab,kw OR ‘income’:ti,ab,kw OR ‘disparit*’:ti,ab,kw OR ‘ineq*’:ti,ab,kw OR [mh “Social Status”] OR ‘caste*’:ti,ab,kw OR ‘vulnerab*’:ti,ab,kw OR [mh “Vulnerability”] OR ‘Education*’:ti,ab,kw OR ‘Literacy’:ti,ab,kw OR ‘housing instability’:ti,ab,kw OR ‘poverty’:ti,ab,kw OR ‘employment’:ti,ab,kw OR ‘Rank’:ti,ab,kw OR ‘achievement’:ti,ab,kw OR ‘indig*’:ti,ab,kw OR ‘Ghetto*’:ti,ab,kw OR ‘Slum*’:ti,ab,kw OR ‘poorness’:ti,ab,kw OR ‘occupation’:ti,ab,kw OR ‘profession’:ti,ab,kw OR ‘job’:ti,ab,kw OR ‘jobs’:ti,ab,kw OR ‘realization*’:ti,ab,kw OR ‘Success’:ti,ab,kw OR ‘qualification’:ti,ab,kw OR [mh “Sociodemographic factors”] OR ‘Sociodemographic*’:ti,ab,kw OR ‘Socio demographic*’:ti,ab,kw OR [mh “Cross-cultural comparison”] OR ‘cultural’:ti,ab,kw OR

‘ethnic’:ti,ab,kw OR ‘racial’:ti,ab,kw OR ‘minorit*’:ti,ab,kw OR [mh “Ethnic and racial minorities”] OR [mh “Minority Groups”] OR [mh “Cultural deprivation”] OR [mh “Cultural diversity”] OR [mh “Cultural characteristics”] OR ‘diversit*’:ti,ab,kw OR [mh “Hierarchy, social”] OR [mh “Social Marginalization”] OR ‘Living condition*’:ti,ab,kw OR ‘Wealth’:ti,ab,kw OR ‘Classism’:ti,ab,kw OR ‘Illiteracy’:ti,ab,kw OR ‘Unemployment’:ti,ab,kw)

Web of Science Core Collection (Clarivate)

(TI=("Lesotho" OR “Maseru” OR "Basutoland" OR "Botswana" OR “Gaborone” OR "Bechuanaland" OR "Kalahari" OR "Namibia" OR “Zambezi” OR “Kavango” OR “Kunene” OR “Omusati” OR “Ohangwena” OR “Oshana” OR “Oshikoto” OR “Omaheke” OR “Otjozondjupa” OR “Erongo” OR “Khomas” OR “Hardap” OR “Karas” OR “Windhoek” OR "Eswatini" OR “Mbabane” OR “Lobamba” OR "Swaziland" OR "Hhohho” OR “Lubombo” OR “Manzini” OR “Shiselweni” OR "South Africa" OR “Cape Town” OR “Eastern Cape” OR “Free State” OR “Gauteng” OR “KwaZulu-Natal” OR “Limpopo” OR “Mpumalanga” OR “Northern Cape” OR “Western Cape” OR "Belgium" OR “Flemish” OR “Walloon” OR “Brussels” OR “Flanders” OR “Wallonia” OR “Dutch” OR “Netherlands” OR "Holland" OR “Amsterdam” OR "Luxemb*" OR “benelux” OR "German*" OR “Berlin” OR "France" OR “Auvergne-Rhône-Alpes” OR “Burgundy-Franche-Comté” OR “Brittany” OR “Center-Val de Loire” OR “Great East” OR “Normandy” OR “New Aquitaine” OR “Occitanie” OR “Pays de la Loire” OR “Provence-Alpes-Côte d'Azur” OR “Paris” OR “French” OR "Monaco" OR "Austria" OR “Vienna” OR "Switzerland" OR “Bern” OR "Liechtenstein" OR “Leichtenstein”) OR AB=("Lesotho" OR “Maseru” OR "Basutoland" OR "Botswana" OR “Gaborone” OR "Bechuanaland" OR "Kalahari" OR "Namibia" OR “Zambezi” OR “Kavango” OR “Kunene” OR “Omusati” OR “Ohangwena” OR “Oshana” OR “Oshikoto” OR “Omaheke” OR “Otjozondjupa” OR “Erongo” OR “Khomas” OR “Hardap” OR “Karas” OR “Windhoek” OR "Eswatini" OR “Mbabane” OR “Lobamba” OR "Swaziland" OR "Hhohho” OR “Lubombo” OR “Manzini” OR “Shiselweni” OR "South Africa" OR “Cape Town” OR “Eastern Cape” OR “Free State” OR “Gauteng” OR “KwaZulu-Natal” OR “Limpopo” OR “Mpumalanga” OR “Northern Cape” OR “Western Cape” OR "Belgium" OR “Flemish” OR “Walloon” OR “Brussels” OR “Flanders” OR “Wallonia” OR “Dutch” OR “Netherlands” OR "Holland" OR “Amsterdam” OR "Luxemb*" OR “benelux” OR "German*" OR “Berlin” OR "France" OR

“Auvergne-Rhône-Alpes” OR “Burgundy-Franche-Comté” OR “Brittany” OR “Center-Val de Loire” OR “Great East” OR “Normandy” OR “New Aquitaine” OR “Occitanie” OR “Pays de la Loire” OR “Provence-Alpes-Côte d'Azur” OR “Paris” OR “French” OR "Monaco" OR "Austria" OR “Vienna” OR "Switzerland" OR “Bern” OR "Liechtenstein" OR “Leichtenstein”)) AND (TI=("initiat*" OR "duration" OR "extent" OR "period" OR "commitment" OR "contin*" OR “Start*” OR “begin*” OR “induct*” OR “endur*” OR “persistence” OR “prolong*” OR “sust*” OR “preserv*” OR “maintaining” OR “engage*”) OR AB=("initiat*" OR "duration" OR "extent" OR "period" OR "commitment" OR "contin*" OR “Start*” OR “begin*” OR “induct*” OR “endur*” OR “persistence” OR “prolong*” OR “sust*” OR “preserv*” OR “maintaining” OR “engage*”)) AND (TI=("breast fe*" OR "breastfe*" OR "milk sharing" OR "wet nursing" OR "human milk" OR "breast milk" OR "maternal milk" OR "mother milk" OR "woman milk" OR "breast pumping*" OR “lactation” OR “Infant Feeding” OR “Maternal Feeding” OR “Child Feeding” OR “Mother’s milk”) OR AB=("breast fe*" OR "breastfe*" OR "milk sharing" OR "wet nursing" OR "human milk" OR "breast milk" OR "maternal milk" OR "mother milk" OR "woman milk" OR "breast pumping*" OR “lactation” OR “Infant Feeding” OR “Maternal Feeding” OR “Child Feeding” OR “Mother’s milk”)) AND (TI=("Factor*" OR "Determinant*" OR "Aspect*" OR "Characteristic*" OR "socioeconomic*" OR "socio economic*” OR “Living standard*” OR “Social*” OR “Economic*” OR "population*" OR "income" OR "disparit*" OR "ineq*" OR "caste*” OR “vulnerab*” OR “Education*” OR “Literacy” OR “housing instability” OR “poverty” OR “employment” OR “Rank” OR “achievement” OR “indig*” OR “Ghetto*” OR “Slum*” OR “poorness” OR “occupation” OR “profession” OR “job” OR “jobs” OR “realization*” OR “Success” OR “qualification” OR “Sociodemographic*” OR “Socio demographic*“ OR “cultural” OR “ethnic” OR “racial” OR “minorit*” OR “diversit*” OR “Living condition*” OR “Wealth” OR “Classism” OR ”Illiteracy” OR “Unemployment”) OR AB=("socioeconomic*" OR "socio economic*” OR “Living standard*” OR “Social*” OR “Economic*” OR "population*" OR "income" OR "disparit*" OR "ineq*" OR "caste*” OR “vulnerab*” OR “Education*” OR “Literacy” OR “housing instability” OR “poverty” OR “employment” OR “Rank” OR “achievement” OR “indig*” OR “Ghetto*” OR “Slum*” OR “poorness” OR “occupation” OR “profession” OR “job” OR “jobs” OR “realization*” OR “Success” OR “qualification” OR “Sociodemographic*” OR “Socio demographic*“ OR “cultural” OR “ethnic” OR “racial” OR “minorit*” OR “diversit*” OR “Living condition*” OR “Wealth” OR “Classism” OR ”Illiteracy” OR “Unemployment”))

Sociology Database (ProQuest)

(MAINSUBJECT.EXACT("South African studies") OR AB,TI("Lesotho" OR “Maseru” OR "Basutoland" OR "Botswana" OR “Gaborone” OR "Bechuanaland" OR "Kalahari" OR "Namibia" OR “Zambezi” OR “Kavango” OR “Kunene” OR “Omusati” OR “Ohangwena” OR “Oshana” OR “Oshikoto” OR “Omaheke” OR “Otjozondjupa” OR “Erongo” OR “Khomas” OR “Hardap” OR “Karas” OR “Windhoek” OR "Eswatini" OR “Mbabane” OR “Lobamba” OR "Swaziland" OR "Hhohho” OR “Lubombo” OR “Manzini” OR “Shiselweni” OR "South Africa" OR “Cape Town” OR “Eastern Cape” OR “Free State” OR “Gauteng” OR “KwaZulu-Natal” OR “Limpopo” OR “Mpumalanga” OR “Northern Cape” OR “Western Cape” OR "Belgium" OR “Flemish” OR “Walloon” OR “Brussels” OR “Flanders” OR “Wallonia” OR “Dutch” OR “Netherlands” OR "Holland" OR “Amsterdam” OR "Luxemb*" OR “benelux” OR "German*" OR “Berlin” OR "France" OR “Auvergne-Rhône-Alpes” OR “Burgundy-Franche-Comté” OR “Brittany” OR “Center-Val de Loire” OR “Great East” OR “Normandy” OR “New Aquitaine” OR “Occitanie” OR “Pays de la Loire” OR “Provence-Alpes-Côte d'Azur” OR “Paris” OR “French” OR "Monaco" OR "Austria" OR “Vienna” OR "Switzerland" OR “Bern” OR "Liechtenstein" OR “Leichtenstein”)) AND (AB,TI("initiat*" OR "duration" OR "extent" OR "period" OR "commitment" OR "contin*" OR “Start*” OR “begin*” OR “induct*” OR “endur*” OR “persistence” OR “prolong*” OR “sust*” OR “preserv*” OR “maintaining” OR “engage*”)) AND (MAINSUBJECT.EXACT("Breastfeeding & lactation”) OR AB,TI("breast fe*" OR "breastfe*" OR "milk sharing" OR "wet nursing" OR "human milk" OR "breast milk" OR "maternal milk" OR "mother milk" OR "woman milk" OR "breast pumping*" OR “lactation” OR “Infant Feeding” OR “Maternal Feeding” OR “Child Feeding” OR “Mother’s milk”)) AND (MAINSUBJECT.EXACT("Socioeconomic status” OR “Socioeconomic factors” OR “Parent socioeconomic status” OR “Parent educational background” OR “Health disparities” OR “Educational attainment” OR “Sociocultural factors” OR “Sociodemographics” OR “Social classes” OR “Poverty” OR “Social privilege” OR “Social exclusion” OR “Health disparities” OR “Access to education” OR “Education” OR “Social privilege” OR “Caste” OR “Health literacy” OR “Health education” OR “Literacy” OR “Employment” OR “Occupations” OR “Indigent care” OR “Ghettos” OR “Minority & ethnic groups” OR “Professions” OR “Ethnicity” OR “Standard of living” OR “Poverty” OR “Income inequality” OR “High income” OR “Income” OR “Economic opportunities” OR “Family income” OR “Personal income” OR “Low income groups” OR “Middle income” OR

“Educational inequality” OR “Academic achievement”) OR AB,TI("socioeconomic*" OR "socio economic*” OR “Living standard*” OR “Social*” OR “Economic*” OR "population*" OR "income" OR "disparit*" OR "ineq*" OR "caste*” OR “vulnerab*” OR “Education*” OR “Literacy” OR “housing instability” OR “poverty” OR “employment” OR “Rank” OR “achievement” OR “indig*” OR “Ghetto*” OR “Slum*” OR “poorness” OR “occupation” OR “profession” OR “job” OR “jobs” OR “realization*” OR “Success” OR “qualification” OR “Sociodemographic*” OR “Socio demographic*“ OR “cultural” OR “ethnic” OR “racial” OR “minorit*” OR “diversit*” OR “Living condition*” OR “Wealth” OR “Classism” OR ”Illiteracy” OR “Unemployment”) OR TI("Factor*" OR "Determinant*" OR "Aspect*" OR "Characteristic*"))

Scopus

(TITLE-ABS("Lesotho" OR “Maseru” OR "Basutoland" OR "Botswana" OR “Gaborone” OR "Bechuanaland" OR "Kalahari" OR "Namibia" OR “Zambezi” OR “Kavango” OR “Kunene” OR “Omusati” OR “Ohangwena” OR “Oshana” OR “Oshikoto” OR “Omaheke” OR “Otjozondjupa” OR “Erongo” OR “Khomas” OR “Hardap” OR “Karas” OR “Windhoek” OR "Eswatini" OR “Mbabane” OR “Lobamba” OR "Swaziland" OR "Hhohho” OR “Lubombo” OR “Manzini” OR “Shiselweni” OR "South Africa" OR “Cape Town” OR “Eastern Cape” OR “Free State” OR “Gauteng” OR “KwaZulu-Natal” OR “Limpopo” OR “Mpumalanga” OR “Northern Cape” OR “Western Cape” OR "Belgium" OR “Flemish” OR “Walloon” OR “Brussels” OR “Flanders” OR “Wallonia” OR “Dutch” OR “Netherlands” OR "Holland" OR “Amsterdam” OR "Luxemb*" OR “benelux” OR "German*" OR “Berlin” OR "France" OR “Auvergne-Rhône-Alpes” OR “Burgundy-Franche-Comté” OR “Brittany” OR “Center-Val de Loire” OR “Great East” OR “Normandy” OR “New Aquitaine” OR “Occitanie” OR “Pays de la Loire” OR “Provence-Alpes-Côte d'Azur” OR “Paris” OR “French” OR "Monaco" OR "Austria" OR “Vienna” OR "Switzerland" OR “Bern” OR "Liechtenstein" OR “Leichtenstein”)) AND (TITLE-ABS("initiat*" OR "duration" OR "extent" OR "period" OR "commitment" OR "contin*" OR “Start*” OR “begin*” OR “induct*” OR “endur*” OR “persistence” OR “prolong*” OR “sust*” OR “preserv*” OR “maintaining” OR “engage*”)) AND (TITLE-ABS(“breast feeding" OR "breastfe*" OR "milk sharing" OR "wet nursing" OR "human milk" OR "breast milk" OR "maternal milk" OR "mother milk" OR "woman milk" OR "breast pumping*" OR “lactation” OR “Infant Feeding” OR “Maternal Feeding” OR “Child

Feeding” OR “Mother s milk”)) AND (TITLE-ABS(“socioeconomic*" OR "socio economic*” OR “Living standard*” OR “Social*” OR “Economic*” OR "population*" OR "income" OR "disparit*" OR "inequity" OR "caste*” OR “vulnerab*” OR “Education*” OR “Literacy” OR “housing instability” OR “poverty” OR “employment” OR “Rank” OR “achievement” OR “indig*” OR “Ghetto*” OR “Slum*” OR “poorness” OR “occupation” OR “profession” OR “job” OR “jobs” OR “realization*” OR “Success” OR “qualification” OR “Sociodemographic*” OR “Socio demographic*“ OR “cultural” OR “ethnic” OR “racial” OR “minorit*” OR “diversit*” OR “Living condition*” OR “Wealth” OR “Classism” OR ”Illiteracy” OR “Unemployment”)) OR (TITLE("Factor*" OR "Determinant*" OR "Aspect*" OR "Characteristic*"))

File S2. Inclusion form

Once you answer no, then exclude

A. Article reported in English or Dutch.

B. Primary study (not case series, case reports, systematic reviews or secondary articles).

C. Article written starting from 2008.

D. The study focuses on one of the following countries: Germany, Liechtenstein, Luxembourg, Austria, Switzerland, Belgium, France, Monaco, The Netherlands, Botswana, Eswatini, Lesotho, Namibia, South Africa

E. Focus on a normal pregnancy and birth. Main focus is not on: preterm birth, woman above 40 years old, complications during pregnancy or birth, maternal chronical illnesses and relevant medical history, multiple gestation, substance abuse (drugs/alcohol/nicotine).

F. Focus on mothers who gave birth starting from 2001.

G. Focus on initiation or duration of breastfeeding.

H. Focus on (a determinant of) the socioeconomic status (SES).

- Income and social protection
- Education
- Unemployment and job insecurity
- Working life conditions
- Food insecurity
- Housing, basic amenities and the environment
- Early childhood development
- Social inclusion and non-discrimination

- Structural conflict

I. Access to affordable health services of decent quality

J. Conclusion: include article for data extraction

File S3. List of tables

Table S1: study characteristics

Included studies	*Studied region*	*Participants*	*Number of participants*	*Inclusion criteria*	*Study design*
Bergman et al. (2016)	Eswatini	Pregnant woman in the peri-urban industrial area Matsapha on the outskirts of the nation's largest city Manzini	1 402	Mother pregnant at time of enrolment, have given birth to a live baby on latest the 31st of March 2015, living in the study area.	Cohort
Bernard et al. (2016)	France	All Catholics and Protestants of Western countries (including France)	NR	NR	Cross-sectional

Betoko et al. (2013)	France	Mothers in France	2 002	Mothers at the maternity wards of Poitiers and Nancy University hospitals between 2003 and 2006. No multiple pregnancies, diabetes history, illiteracy and moving outside the region planned in the next three years. Women aged 18–45 years.	Cohort
Bonet et al. (2012)	France	Mothers in France	2 002	Mothers at the maternity wards of Poitiers and Nancy University hospitals between 2003 and 2006. No multiple pregnancies, diabetes history, illiteracy and moving outside the region planned in the next three years. Women aged 18–45 years.	Cohort
Bournez et al. (2017)	France	Infants born in France in 2011	10 931	Children born in 2011 and after 33 weeks of amenorrhoea. Mothers aged ≥18 years who were not planning to move outside Metropolitan France in the next 3 years. Mothers able to read French, Arabic, Turkish, or English, the most	Cohort

				common languages spoken by women giving birth in France. Also twins.	
Brenne et al. (2018)	Germany	Mothers in Berlin	6 884	Mothers who were admitted to one of the three participating maternity clinics in Berlin for the birth of their child and in whom the child was born with signs of life and after 24 weeks of pregnancy. Study participants were at least 18 years old when their child was born, permanently resident in Germany.	Cohort
Bürger et al. (2022)	Austria	Mothers in Austria	1 214	Mothers of legal age. Mothers in maternity wards.	Cohort
Camier et al. (2020)	France	Infants born in France in 2011	7 556	Singleton or twins born after 33 weeks in France. Mothers aged 18 years or older. Not planning to move outside metropolitan France	Cohort

				in the next 3 years. No babies with specific dietary issues.	
Castetbon et al. (2020)	France	Mother – infant dyads in France	1 487	Mothers aged 18 or over, not living in an institution, French-speaking or with access to help filling out questionnaires, gestational age at delivery ≥33 amenorrhea weeks and no severe newborn pathology that required transfer to a specialized neonatology unit just after delivery. Also twins.	Cohort
Courtois et al. (2021)	France	French parous women	29 953	No nulliparous women. Woman aged ≥18 years.	Cohort
Frenoy et al. (2021)	France	Homeless families in France with children	456	Families had to include at least one parent, have at least one child older than 6 months and younger than 5 years, speak one of the 17 languages considered in the survey and be able	Cross-sectional

				to provide informed consent. Living in different kinds of shelters.	
Girard et al. (2016)	France	Mothers in France	1 891	Pregnant women who were 18 years or older and less than 24 weeks of gestation. No diagnoses of diabetes, not expecting multiple births, able to read/write in French, not planning on moving outside the area within the next three years.	Cohort
Goosen et al. (2014)	South Africa	Primary caregivers of infants in the Avian Park and Zwelethemba communities in Worcester, Western Cape	108	Infants under 6 months of age. Spoke isiXhosa, Afrikaans, or English. Lived in the area for at least 5 years.	Cross-sectioneel

Guajardo-Villar et al. (2022)	France	Mothers in France	12 224 (2010)<br><br>11 089 (2016)	All births with a gestational age of at least 22 weeks of amenorrhoea and a weight of at least 500g in all maternity units. Including children born alive outside maternity units who were subsequently transferred to one. Mothers older than 18 years. Also twins.	Cohort
Hentges and Pilot (2021)	The Netherlands	Female academic staff (mothers) from 14 Dutch research universities.	13	Academic employees who are mothers. Can be breastfeeding experts, such as lactation consultants and researchers. Both native Dutch and non-Dutch women. Healthy mothers with healthy children. Not on maternity leave. Currently BF or have breastfed/pumped at work in the past five years. Both full-time and part-time employees. Good command of English.	Qualitative research
Horwood et al. (2019)	South Africa	Mothers working in the informal economy in an	247	Mothers aged 18 years or older. In informal work for at least 3 months. Working at least 10 hours per week. Mother of a living child under	Cross-sectional

		urban and a rural site in KwaZulu-Natal		2 years. Able to communicate in a local language with researchers.	
Horwood et al. (2018)	South Africa	Caregivers in KwaZulu-Natal	4 059	All caregivers aged 15 years or above attending fixed and mobile PHC's in KwaZulu-Natal with an infant aged 13-16 weeks (91–111 days) were eligible to participate, including maternal and non-maternal caregivers.	Cross-sectional
Horwood et al. (2020)	South Africa	Informal workers (mothers) in one urban and one rural site in KwaZulu-Natal	NR	Woman having a child under five years, working in an informal job, living in the study area. Participants were eligible to participate if they were 18 years or older and had been working in the informal economy for more than 6 months.	Qualitative research

Hunter-Adams et al. (2016)	South Africa	Somali, Congolese, and Zimbabwean cross-border migrants in Cape Town within the past 5 years	23	Women over 18, pregnant or gave birth in the last 2 years, self-identified as Somali, Congolese, or Zimbabwean.	Qualitative research
Ikobah et al. (2023)	South Africa	BF mothers in Western Cape	110	Mothers of infants aged six weeks to one year that visited the children emergency unit of the Department of Paediatrics and Child Health of Tygerberg Academic Hospital, Cape Town for minor illnesses between May 2017 and June 2017.	Cross-sectional
Kersuzan et al. (2018)	France	Infants born in France in 2011	14 110	Legal age, singleton or twins, delivered after at least 33 weeks of amenorrhoea, not be planning to live outside metropolitan France during the following three years. Speaks French, English, Arabic or Turkish.	Cohort

Kohlhuber et al. (2008)	Germany	Mothers who delivered in the state of Bavaria in April 2005	3 822	Woman older than 18 years old. Also twins.	Cohort
Kyei et al. (2014)	South Africa	BF mothers in the Vhembe district of Limpopo Province	2 660	Woman between the age of 13 and 50 years.	Cohort
Lange et al. (2016)	Germany	Neonates in Pomerania	6 828 babies and 6 747 mothers	Singletons or twins.	Cohort
Libuda et al. (2013)	Germany	Mothers in the access panel of Munich	1 804	Children from between 5 and 36 months old.	RCT
Logan et al. (2016)	Germany	Newborns and mothers in Germany	1 090 newborns of 1066 mothers (Cohort 1)	Study populations restricted to singleton term newborns (≥37 weeks). No participants with outpatient delivery or immediate transfer to intensive care. Maternal age more than 18 years.	Cohort

			1 006 newborns of 970 mothers (Cohort 2)	Sufficient language proficiency in German, Turkish, or Russian.	
Luthuli et al. (2020)	South Africa	Informal workers between 32 and 38 weeks pregnant in Durban	24	Pregnant informal workers aged 18 years or older. Visited one of the two peri-urban PHC's in Durban. Last trimester of pregnancy between 32 and 38 weeks pregnant. Woman in informal work for more than 6 months, woman working less then 3 days a week. Woman who did not plan to leave the area after delivery.	Cohort
Mbhenyane et al. (2023)	South Africa	Mother-baby pairs in Mopani and Vhembe districts, Limpopo Province	169	NR	Cross-sectional

Motadi et al. (2019)	South Africa	Mothers of children from a rural area in the Limpopo province	360	Children younger than 2 years. Mothers who had breastfed their children during the first 2 years of life. No non-parent caregivers.	Cross-sectional
Mphasha et al. (2023)	South Africa	Caregivers of infants in Seshego	86	Caregivers of infants aged 0 to 24 months. Must be able to speak Sepedi or English. Must be at least 18 years old.	Cross-sectional
Ndirangu et al. (2018)	Namibia	Households and mothers in Namibia	6 849 households and 7 308 women (2000)<br><br>9 970 households and 10 352 women (2006 to 2007)<br><br>11 004 households and 9	Mothers aged 15 to 49 years.	Cohort

			940 women (2013)		
Oberfichtner et al. (2023)	Austria	Primiparous women delivering infants at Kepler University Hospital	140	Primiparous women delivering infants between 38 and 42 weeks of gestation. No prenatal or postpartum diagnoses restricting EBF, no chronic illnesses, no long-term medication, no prior breast surgery limiting unrestricted BF. Good knowledge of German or English.	Cohort
Quittner et al. (2017)	The Netherlands	All infants in Amsterdam born between 2009 and 2015	75 543	NR	Cross-sectional
Rasenack et al. (2012)	Germany	Women who had recently given birth in Freiburg	433	Women given birth to a living child between August and December 2007 at three specific facilities: University Gynaecological Clinic, Evangelical Diakonie Hospital, Geburtshaus Mayenrain in Freiburg. No multiple births.	Cohort

				Woman aged above 18 years old. Good knowledge of German. Also preterm births included.	
Rebhan et al. (2009)	Germany	Mothers who delivered a baby in April 2005 in Bavaria	3 103	Woman older than 18 years old. Also children with low birth weight included.	Cohort
Robert et al. (2014a)	Belgium	Families of children living in Wallonia in 2012	525 respondents: 480 mothers, 37 fathers and 8 grandmothers	Children born in 2010. Children aged 18–24 months. Families with mothers who breastfed at birth.	Cohort
Robert et al. (2014b)	Belgium	Children born in Wallonia and the Brussels-Capital Region	525 children in Wallonia and 544 children in Brussels	Children born in 2010. Children registered as residents in either Brussels or Wallonia. Mothers who breastfed at the maternity ward. Good knowledge of the language.	Cross-sectional

Seabela et al. (2023)	South Africa	Mothers in Mpumalanga	30	Mothers aged 18 - 42 years. Children under the age of two years which visited the PHC in Ermelo (a town in the Mpumalanga). Had ever breastfed or were breastfeeding at the time of the study.	Qualitative research
Silva et al. (2012)	The Netherlands	Mothers with a Dutch ethnicity in Rotterdam	2 972 mothers and their children	Children born between April 2002 and January 2006. No twins, no second or third child of the same mother, no missing maternal education.	Cohort
Smuts et al. (2008)	South Africa	Children and their caregivers in rural districts of the Eastern Cape and KwaZulu-Natal provinces	3 782	0- to 71-month-old children.	Cross-sectional
Stumbitz and Jaga (2020)	South Africa	Low paid mothers and managers in Cape Town	36 mothers and 15 managers	Child born after 2011. Mothers following South Africa's adoption of the Tshwane Declaration of Support for Breastfeeding, which experienced	Qualitative research

				working during pregnancy and returned to work after maternity leave.	
Swarts et al. (2010)	South Africa	Mothers in the lower Umfolozi district war memorial hospital, KwaZulu-Natal	100	NR	Cross-sectional
Van Der Bijl et al. (2023)	South Africa	Doctor mothers in Bloemfontein	104 mothers and 132 children	Doctor mothers with a medical or equivalent degree working full or part-time in the government or private sector, with biological children under 5 years.	Cohort
Van Rossem et al. (2009a)	The Netherlands	Dutch mothers living in Rotterdam	2 914	Expected delivery data between April 2002 and January 2006. Mothers who lives in the study area with Dutch ethnicity. No twin pregnancies, second or third pregnancy.	Cohort

Van Rossem et al. (2009b)	The Netherlands	Breastfeeding mothers in ethnic minority groups In Rotterdam	3 848	Expected delivery data between April 2002 and January 2006. Mothers who lives in the study area with Dutch ethnicity. No twin pregnancies, second or third pregnancy.	Cohort
Wagner et al. (2019)	France	Infants born in France in 2011	13 774 mother-infant dyads	Infants born after 33 weeks' gestation. Mother's age 18 years or older. Mother not planning to move outside of France in the next 3 years. Foreign families could participate if mothers could read French, Arabic, Turkish, or English. No twin (one twin randomly selected from each pair).	Cohort
Walburg et al. (2010)	France and Germany	First-time mothers in Toulouse (France) and Hamburg, Köln, Bochum, Aachen, Kaiserslautern,	126 French women and 80 German women	All participants were first-time mothers fluent in the language of the country.	Cohort

	Berlin, and Düsseldorf (all from West Germany)	

Abbreviations: BF: Breastfeeding, EBF: Exclusive Breasfteeding, NR: Not Reported, PHC: Primary Health Care Clinic

Table S2: Quality Assessment: all performed with NOS, except for Hentges and Pilot (2021), Horwood et al. (2020) Hunter-Adams et al. (2016), Seabela et al. (2023) and Stumbitz and Jaga (2020), they are performed with QARI; Libuda et al. (2013) is performed with RoB2.

Included studies	*Score*
Bergman et al. (2016)	Good study ******** (8 stars)
Bernard et al. (2016)	Good study ******** (8 stars)
Betoko et al. (2013)	Good study ******* (7 stars)
Bonet et al. (2012)	Good study ******** (8 stars)
Bournez et al. (2017)	Good study ******* (7 stars)
Brenne et al. (2018)	Good study ******** (8 stars)
Bürger et al. (2022)	Good study ******* (7 stars)
Camier et al. (2020)	Good study ******** (8 stars)
Castetbon et al. (2020)	Good study ******** (8 stars)
Courtois et al. (2021)	Satisfactory study ****** (6 stars)
Frenoy et al. (2021)	Very good study ********* (9 stars)

Girard et al. (2016)	Good study ******** (8 stars)
Goosen et al. (2014)	Good study ******** (8 stars)
Guajardo-Villar et al. (2022)	Good study ******** (8 stars)
Hentges and Pilot (2021)	Good study
Horwood et al. (2019)	Good study ******** (8 stars)
Horwood et al. (2018)	Very good study ********* (9 stars)
Horwood et al. (2020)	Good study
Hunter-Adams et al. (2016)	Good study
Ikobah et al. (2023)	Good study ******** (8 stars)
Kersuzan et al. (2018)	Good study ******** (8 stars)
Kohlhuber et al. (2008)	Good study ******** (8 stars)
Kyei et al. (2014)	Good study ******* (7 stars)
Lange et al. (2016)	Good study ******** (8 stars)

Libuda et al. (2013)	Good study
Logan et al. (2016)	Good study ******** (8 stars)
Luthuli et al. (2020)	Good study ******** (8 stars)
Mbhenyane et al. (2023)	Good study ******** (8 stars)
Motadi et al. (2019)	Good study ******** (8 stars)
Mphasha et al. (2023)	Very good study ********* (9 stars)
Ndirangu et al. (2018)	Satisfactory study ****** (6 stars)
Oberfichtner et al. (2023)	Good study ******* (7 stars)
Quittner et al. (2017)	Very good study ********* (9 stars)
Rasenack et al. (2012)	Good study ******** (8 stars)
Rebhan et al. (2009)	Satisfactory study ****** (6 stars)
Robert et al. (2014a)	Satisfactory study ****** (6 stars)
Robert et al. (2014b)	Very good study ********* (9 stars)

Seabela et al. (2023)	Good study
Silva et al. (2012)	Satisfactory study ****** (6 stars)
Smuts et al. (2008)	Very good study ********* (9 stars)
Stumbitz and Jaga (2020)	Good study
Swarts et al. (2010)	Good study ******* (7 stars)
Van Der Bijl et al. (2023)	Good study ******* (7 stars)
Van Rossem et al. (2009a)	Satisfactory study ****** (6 stars)
Van Rossem et al. (2009b)	Good study ******** (8 stars)
Wagner et al. (2019)	Good study ******** (8 stars)
Walburg et al. (2010)	Good study ******* (7 stars)

Abbreviations: NOS: Newcastle-Ottowa Scale (Wells et al. (2021)), QARI: Qualitative Assessment and Review Instrument (Joanna Briggs Institute (2014)), RoB2: The Risk of Bias 2 Tool (Sterne et al. (2019)).

Table S3.1: Educational level, breastfeeding initiation, Western Europe

Included studies	*Impact of* ***educational level on breastfeeding initiation***
Betoko et al. (2013)	NR
Bonet et al. (2013)	NR
Bournez et al. (2017)	NR
Bürger et al. (2022)	NR
Camier et al. (2020)	Maternal educational level positively related to higher BF rates.
Courtois et al. (2021)	BF mothers had a higher educational level compared to non-BF mothers.
Frenoy et al. (2021)	NR
Girard et al. (2016)	Mothers with a university degree were more likely to breastfeed. Higher levels of education increased the odds of BF initiation. . In the category “no diploma”, 55% initiated BF. In the category ‘university degree’, 83,4% initiated BF.
Guajardo-Villar et al. (2022)	Exclusive BF initiation rates varied greatly by education level: the lower the level the lower the rate. Compared with mothers with more than 2 years of university education, mothers with 1–2 years of university education and those with no or primary level education had lower rates. Mixed BF initiation rates did not vary greatly according to education level. Both years combined, exclusive and any BF rates were lowest in mothers with no or primary level education. Education level was one of the individual-level covariates that had the strongest

	association with BF initiation. Specifically, as the mother's level of education decreased, the exclusive BF initiation rates decreased substantially and the mixed rate decreased slightly, resulting in a net decrease in any BF initiation rates.
Kersuzan et al. (2018)	Less mothers in the ‘lower than secondary school’ group initiated BF after birth than in the ‘higher education’ group.
Kohlhuber et al. (2008)	Mothers with <10 years of education were more likely not to breastfeed. Participants with 10–11 years of education had a higher risk not to breastfeed than women with >11 years of education.
Lange et al. (2017)	Factors positively influencing EBF intention included higher education.
Logan et al. (2016)	Increased rates of BF initiation and duration observed over an 11-year period among mothers with more than 12 years of education, while no significant change in BF behavior was observed among less educated mothers.
Oberfichtner et al. (2023)	High prevalence of BF is associated with a high educational level.
Rasenack et al. (2012)	NR
Rebhan et al. (2009)	NR
Robert et al. (2014b)	NR
Silva et al. (2012)	Children of low-educated mothers were less likely to be breastfed compared to children of high-educated mothers.
Van Rossem et al. (2009a)	89.1% (2596 of 2914) of the women started BF, which was lower for mothers in education category 1 (low education) (73.1%) than for mothers in education category 4 (high education) (95.5%).

Abbreviations; BF: Breastfeeding, EBF: Exclusive Breastfeeding, NR: Not Reported

Table S3.2: Educational level, breastfeeding initiation, Southern Africa

Included studies	*Impact of **educational level on breastfeeding initiation***
Bergman et al. (2016)	NR
Horwood et al. (2018)	Most of the parents completed grades 8 to 11 (so they went to school until 14 to 18 years). Less than half (43.4%) of the mothers reported that they had initiated BF within one hour of delivery.
Ikobah et al. (2023)	Most parents were generally well-educated with secondary school or higher educational qualifications. Timely initiation of BF within one hour of life occurred in 79 (72.4%) of the infants, and 83 (76.1%) were ever breastfed.
Kyei et al. (2014)	NR
Ndirangu et al. (2018)	EIBF among mothers with a secondary education increased from 39.9% (95% CI: 36.4–43.4) in 2000 to 50% (95% CI: 47.1–53.0) in 2013. Moreover, maternal age as a determinant of EIBF is largely dependent on the presence of factors such education level.
Swarts et al. (2010)	72% of women chose BF as first infant feeding method. The high number of women choosing BF may probably also be linked to the fact that the majority had a high school education (86% of the study population went to high school).

Abbreviations; BF: Breastfeeding, NR: Not Reported

Table S3.3: Educational level, breastfeeding duration, Western Europe

Included studies	*Impact of* ***educational level on breastfeeding duration***
Betoko et al. (2013)	Mothers of infants with high scores on pattern-2 (= longer breastfeeding, late CF introduction) were more likely to have high education level.
Bonet et al. (2013)	Breastfeeding rates at 4 months of infant's age (both any and almost exclusive breastfeeding rates) were higher among those with a higher educational level (women were more likely to continue any or almost exclusive breastfeeding if they had a university degree).
Bournez et al. (2017)	Maternal education level impacted the age of CF introduction, with mothers having less than 9 years of schooling more likely to introduce CF before 4 months.
Bürger et al. (2022)	Factors positively influencing breastfeeding maintenance up to 12 months or longer include higher education.
Camier et al. (2020)	Maternal education level: Positively related to patterns involving later CF initiation and longer BF duration.
Courtois et al. (2021)	NR
Frenoy et al. (2021)	Education level affected extended breastfeeding: for instance, sub-Saharan African mothers without a high school diploma breastfed longer than their non-African counterparts, whereas high school graduates born elsewhere in Africa breastfed longer. However, no significant difference was observed in breastfeeding duration based on maternal birth region among mothers with tertiary education.
Girard et al. (2016)	NR
Guajardo-Villar et al. (2022)	NR

Kersuzan et al. (2018)	Less mothers in the ‘lower than secondary school’ group continued breastfeeding up to at least 6 months.
Kohlhuber et al. (2008)	Mothers with lower education breastfed for less time than older mothers with higher educational level.
Lange et al. (2017)	Lower education was associated with shorter breastfeeding duration.
Logan et al. (2016)	Increased rates of breastfeeding duration observed over an 11-year period among mothers with more than 12 years of education, while no significant change in breastfeeding behaviour was observed among less educated mothers.
Oberfichtner et al. (2023)	Women with a high-school diploma ceased breastfeeding signifcantly more often after more than 6 months. Long duration of breastfeeding is associated with a high educational level. The present study also found that women with university entrance qualifcations or higher educational status were signifcantly more likely to cease breastfeeding only after more than 6 months.
Rasenack et al. (2012)	Women with the qualification to undertake university studies breastfeed significantly longer than women with a secondary school education or women not completing their schooling.
Rebhan et al. (2009)	Risk factors for early introduction of complementary food (all foods other than human milk or formula milk) were low level of education.
Robert et al. (2014b)	In both Brussels and Wallonia, PIM as a weaning reason at less than 6 months was mentioned more often among women who had less education.
Silva et al. (2012)	NR

Van Rossem et al. (2009a)	An effect was seen in the association between educational level and continuing breastfeeding for 2 months. During this period (mothers who continued breastfeeding from birth op to two months), more mothers in education category 4 (high: higher academic education) than mothers in education category 1 (low: no education, or primary school: <3 years of general secondary school) continued breastfeeding and more mothers in education category 4 continued exclusive breastfeeding (OR: 2.78 [95% CI: 2.04–3.80]). The continuation of breastfeeding between 2 and 6 months did not differ between mothers in education category 4 and education category 1 (OR: 1.17 [95% CI: 0.79–1.72]). At 6 months, 39.3% (405 of 1031) of the highest-educated mothers and 15.2% (53 of 349) of the lowest-educated mothers were still breastfeeding.

Abbreviations; CF: Complementary Feeding, NR: Not Reported, PIM: Perceived Insufficient Milk

Table S3.4: Educational level, breastfeeding duration, Southern Africa

Included studies	*Impact of* ***educational level on breastfeeding duration***
Bergman et al. (2016)	74% did have at least one year of secondary education. The probability to practice EBF for 6 months was estimated to around 50%.
Horwood et al. (2018)	NR
Ikobah et al. (2023)	NR
Kyei et al. (2014)	There was no relationship between breastfeeding in months and educational level.
Ndirangu et al. (2018)	NR
Swarts et al. (2010)	NR

Abbreviations; EBF: Exclusive Breastfeeding, NR: Not Reported

Table S3.5; Employment, income and the work environment; breastfeeding initiation, Western Europe

Included studies	*Impact of* ***employment, income and the work environment on breastfeeding initiation***
Bonet et al. (2012)	High rates of any breastfeeding were observed among both low- and high-income women who did not return to work before 4 months postpartum. However, almost exclusive breastfeeding was more common among low-income women. Maternal employment at birth did not significantly affect breastfeeding initiation after accounting for other factors.
Bournez et al. (2017)	NR
Bürger et al. (2022)	NR
Camier et al. (2020)	NR
Castetbon et al. (2020)	The study found that the RTW time was similar for women who did not breastfeed and those who breastfed for less than four months, suggesting that breastfeeding initiation is not solely dependent on planned RTW. Women who exclusively breastfed or mixed fed at birth also had similar RTW times.
Courtois et al. (2021)	NR
Girard et al. (2016)	Mother's return to work was not a significant determinant. Family income was not a significant determinant.
Guajardo-Villar et al. (2022)	For mothers with a household income above €3000 per month, the decrease in exclusive breastfeeding initiation was lower than average, as was the increase in mixed breastfeeding initiation. Conversely, mothers with an income below €1500 per month saw a greater-than-average increase in mixed breastfeeding initiation,

	leading to a smaller reduction in overall breastfeeding initiation. Exclusive breastfeeding initiation dropped more sharply among the lowest-income mothers, highlighting growing socio-demographic inequalities from 2010 to 2016. Mixed breastfeeding initiation increased most in low-income households, while any breastfeeding initiation decreased most among the highest-income mothers.
Hentges and Pilot (2021)	NR
Kersuzan et al. (2018)	NR
Lange et al. (2016)	Factors positively influencing exclusive breastfeeding intention included higher income. A lack of breastfeeding intention was important as a major risk factor for refusing breastfeeding initiation.
Logan et al. (2016)	NR
Robert et al. (2014a)	NR
Robert et al. (2014b)	NR
Rasenack et al. (2012)	NR
Walburg et al. (2010)	NR

Abbreviations; NR: Not Reported, RTW: Return To Work

Table S3.6: employment, income and the work environment; breastfeeding initation; Southern Africa

Included studies	*Impact of* ***employment, income and the work environment on breastfeeding initiation***
Bergman et al. (2016)	NR
Goosen et al. (2014)	NR
Horwood et al. (2019)	NR
Horwood et al. (2018)	NR
Horwood et al. (2020)	NR
Hunter-Adams et al. (2016)	NR
Kyei et al. (2014)	There was no relationship found between breastfeeding and employment status.
Luthuli et al. (2020)	NR
Mbhenyane et al. (2023)	The study found that mothers in the BFHI group had a significantly higher income compared to those in the non-BFHI group. Interestingly, mothers who attended non-baby-friendly facilities exhibited better breastfeeding practices. But mothers who attended baby-friendly hospitals were more likely to initiate breastfeeding.

Motadi et al. (2019)	NR
Ndirangu et al. (2018)	EIBF was significantly associated with wealth. Multivariate analysis revealed that mothers from households with a poorer wealth index had 82% higher odds of practicing EIBF compared to those from wealthier households.
Seabela et al. (2023)	NR
Stumbitz and Jaga (2020)	NR
Swarts et al. (2010)	Only 4% cited full-time work or study as the reason for choosing formula feeding due to inability to stay home with their infants.
Van Der Bijl et al. (2023)	NR

Abbreviations; BFHI: Baby Friendly Hospital Initiative, NR: Not Reported

Table S3.7; employment, income and the work environment; breastfeeding duration, Western Europe

Included studies	*Impact of* ***employment, income and the work environment on breastfeeding duration***
Bonet et al. (2012)	NR
Bournez et al. (2017)	Factors like occupation type and monthly income showed significance in bivariate analysis for complementary feeding practices, not in multivariable analysis. Maternal factors are more strongly associated with complementary feeding introduction age than paternal factors.
Bürger et al. (2022)	Mothers who returned to work within the first year after childbirth were less likely to continue breastfeeding at 12 months compared to those who did not return to work, indicating a significant impact of employment on breastfeeding duration. Additionally, income level played a crucial role, with higher income associated with longer exclusive breastfeeding duration. A household income below €2500 heightened the risk of introducing infant formula by approximately 80%. Despite extensive maternity leave and benefits in Austria, the likelihood of breastfeeding at 12 months decreased for mothers returning to work within the first year. Further research on workplace interventions and nursing leave utilization could shed more light on the importance of breastfeeding-friendly work environments for overall breastfeeding duration.
Camier et al. (2020)	Wealthier families are less likely to practice prolonged breastfeeding and early introduction of food pieces. Overall, higher income was associated with healthier feeding practices. Returning to work later is linked to longer breastfeeding.
Castetbon et al. (2020)	Women who breastfed for over 4 months had a longer return-to-work time of 198 days, delayed by 31% after adjustments. Employment type played a significant role in RTW time, with flexible working hours and certain occupations facilitating continued breastfeeding. Self-employed women and those in flexible

	jobs were more likely to sustain breastfeeding. While returning to work didn't halt breastfeeding continuation, favourable work conditions and job types were vital factors.
Courtois et al. (2021)	Returning to work was not a major factor for breastfeeding cessation.
Girard et al. (2016)	Mother's return to work was not a significant determinant. Family income was not a significant determinant.
Guajardo-Villar et al. (2022)	NR
Hentges and Pilot (2021)	Extending leave beyond the statutory 10 weeks led to longer breastfeeding durations. Many faced difficulties booking lactation rooms due to overlapping schedules, with some universities having only one room for multiple users. Alternative spaces like meeting or storage rooms were sometimes used, though concerns over hygiene and facilities were common. Support varied by work culture, with female-dominated departments being more supportive. On-campus childcare was seen as beneficial for breastfeeding, but academic schedules often clashed with lactation breaks. Teaching positions were less flexible than research roles.
Kersuzan et al. (2018)	Planning an early return to work negatively affects breastfeeding continuation more than the choice of feeding method.
Lange et al. (2016)	NR
Logan et al. (2016)	Returning to work did not affect breastfeeding cessation at any time.
Robert et al. (2014a)	Higher household income was associated with longer exclusive breastfeeding, with children from families earning over 2000 euros significantly less likely to stop being exclusively breastfed. Early return to work negatively impacted

	breastfeeding duration, as mothers who returned to work within 3 months after birth were less likely to cease any breastfeeding and exclusive breastfeeding compared to others. In the study, 21% of women returned to work at 3 months or earlier, while over 45% extended their maternity leave with parental leave.
Robert et al. (2014b)	"Back to work" became the primary reason for stopping breastfeeding beyond three months after birth in Wallonia.
Rasenack et al. (2012)	Reasons for stopping breastfeeding: employment (5%).
Walburg et al. (2010)	Reasons for breastfeeding cessation for German women: RTW (27%).<br>Reasons for breastfeeding cessation for French women: RTW (19%).

Abbreviations; NR: Not reported, RTW: Return to Work

Table S3.8; Employment, income and the work environment; breastfeeding duration; Southern Africa

Included studies	*Impact of* ***employment, income and the work environment on breastfeeding duration***
Bergman et al. (2016)	Mothers without jobs were 55% less likely to stop exclusive breastfeeding before six months, indicating the need to return to work significantly impacts breastfeeding duration. Despite this, high unemployment rates lead many women to prioritize work over breastfeeding.
Goosen et al. (2014)	Employment status potentially affecting the ability to exclusively breastfeed due to work constraints.
Horwood et al. (2019)	Breastfeeding was disrupted for many due to the need to return to work, affecting 35.1% of all mothers, including 40.5% of domestic workers and 31.5% of informal traders. There is a belief that informal workers, with their flexible working hours, may be more likely to breastfeed. Most women in the informal economy have been in their jobs for several years, earning less than R1000 ($80) per month despite working long hours and often in the same location daily. They face tough conditions, such as no pay during illness and inadequate shelter when working outdoors. Children not taken to work are typically cared for by family members, mainly grandmothers. Domestic workers tend to have better working conditions and receive better pay and benefits. However, they are less likely to bring their children to work, indicating different childcare challenges.
Horwood et al. (2018)	The following factor was associated with not breastfeeding at week 14: richer households and having returned at work. Being in the highest socio-economic tertile was a risk factors for not exclusively breastfeeding, returning to work and school was associated with less exclusive breastfeeding.
Horwood et al. (2020)	Mothers breastfed because it is cheaper than formula feeding. Workplaces were seen as unsafe and unhygienic for breastfeeding and childcare, forcing early returns to work for financial reasons. Most women initiated breastfeeding, but returning to work shortened breastfeeding duration.

Hunter-Adams et al. (2016)	Participants identified low breastfeeding rates as being influenced by household and formal work. Employment status affected their access to resources and social support, contributing to stress and impacting their ability to breastfeed.
Kyei et al. (2014)	There was no relationship found between breastfeeding and employment status.
Luthuli et al. (2020)	Participants relied on savings and child support grants while not working. Half returned between one and two months, often earlier than planned due to financial strain. Returning to work was a key reason for stopping breastfeeding.
Mbhenyane et al. (2023)	The study found that mothers in the BFHI group had a significantly higher income compared to those in the non-BFHI group. Interestingly, mothers who attended non-baby-friendly facilities exhibited better breastfeeding practices.
Motadi et al. (2019)	A small percentage (3.9%) cited going back to school or work as the reason for stopping breastfeeding.
Ndirangu et al. (2018)	NR
Seabela et al. (2023)	Mothers who were employed in this study, mentioned work-related issues hindering breastfeeding, such as working overtime and shifts (i.e., workloads), as well as the inability to get maternity leave and working at a place that does not have space to accommodate babies.
Stumbitz and Jaga (2020)	Most mothers lacked financial support from the babies' fathers, forcing them to return to work early. Low-income mothers were often unaware of their rights to workplace breastfeeding support or felt breastfeeding was a lower priority amid daily struggles. This inequality affected breastfeeding practices and the ability to meet the WHO's six-month exclusive breastfeeding goal. Both employers and low-income mothers viewed breastfeeding as a private issue, not a workplace concern. Mothers lacked confidence to discuss expressing breastmilk at work.

Swarts et al. (2010)	Only 4% cited full-time work or study as the reason for choosing formula feeding due to inability to stay home with their infants.
Van Der Bijl et al. (2023)	55% felt that being a doctor influenced their breastfeeding duration, with career advancement and work-related stress being significant factors.

Abbreviations; BFHI: Baby Friendly Hospital Initiative, NR: Not Reported

Table S3.9; Housing, basic amenities and the environment; breastfeeding initiation, Western Europe

Included studies	*Impact of* ***housing, basic amenities and the environment on breastfeeding initiation***
Courtois et al. (2021)	Area of residence varied between breastfeeding and non-breastfeeding groups.
Frenoy et al. (2021)	The study found that breastfeeding rates were relatively high in homeless populations, with 86% initiating breastfeeding.
Libuda et al. (2013)	Regional differences in breastfeeding initiation: more breastfeeding in East Germany in comparison with West Germany and also more in north Germany in comparison to south German.

Table S3.10; Housing, basic amenities and the environment; breastfeeding initiation; Southern Africa

Included studies	*Impact of* ***housing, basic amenities and the environment on breastfeeding initiation***
Bergman et al. (2016)	NR
Horwood et al. (2019)	There were significant differences in the household environment between the two groups with domestic workers having better access to safe water and sanitation in their homes compared to informal traders. Domestic workers often living in the homes of their employers and receiving benefits.
Horwood et al. (2018)	NR
Horwood et al. (2020)	Mothers in South Africa, raised hygiene concerns with expressed milk. This was coupled with the lack of infrastructure to support it, such as a fridge at home. Given the poor sanitation in low-income settlements where many women lived, they were concerned the expressed milk might become contaminated.
Hunter-Adams et al. (2016)	Participants mainly resided in shared accommodations like larger houses, apartments, or subdivided warehouses. Participants living in overcrowded and substandard housing conditions in Cape Town felt that these living conditions were stressful and contributed to their perception that breastfeeding was impractical.
Kyei et al. (2014)	NR
Ndirangu et al. (2018)	Mothers in rural areas had 42% reduced odds of early initiation of breastfeeding compared with urban.
Smuts et al. (2008)	Toilet facilities (31 to 96%), tap water (9 to 38%), electricity (8 to 51%) varied among provinces. A large proportion of the study population did not have access to basic services. Differences were observed within and between provinces. Results: for infants younger than six months, more than 80% were breast-feeding.

Stumbitz and Jaga (2020)	A lack of a safe environment, hindered access to public transport, and high levels, of stress from crime, gender-based violence and poverty has an impact on these women's capacity to pay attention to their lactating bodies.
Swarts et al. (2010)	The majority of the women in this study came from a poor socio-economic background and they would not be able to formula feed their infants safely and sustainably. Many of these infants of mothers who did not have access to water and flush toilets may then get diarrhea, infections and malnutrition that may even lead to death. Results: 72% of women who chose breastfeeding as first infant feeding method.

Abbreviations; NR: Not Reported

Table S3.11; Housing, basic amenities and the environment, breastfeeding duration, Western Europe

Included studies	*Impact of **housing, basic amenities and the environment on breastfeeding duration***
Courtois et al. (2021)	Area of residence varied between breastfeeding and non-breastfeeding groups.
Frenoy et al. (2021)	The study found that breastfeeding rates were relatively high in homeless populations, with 59% continuing for 6 months or more.
Libuda et al. (2013)	Higher rates of exclusive breastfeeding at the age of 4 months in those mothers living in small in comparison with large communities.

Table S3.12; Housing, basic amenities and the environment; breastfeeding duration, Southern Africa

Included studies	*Impact of* ***housing, basic amenities and the environment on breastfeeding initiation***
Bergman et al. (2016)	A total of 75% of the mothers shared a toilet with people outside their household, access to water was better with 69 % having a tap on site. It was very common to rent housing 74 % and by 71% the place only had one room. Socially vulnerable mothers had a small decrease in probability to discontinue exclusive breastfeeding.
Horwood et al. (2019)	There were significant differences in the household environment between the two groups with domestic workers having better access to safe water and sanitation in their homes compared to informal traders. Domestic workers often living in the homes of their employers and receiving benefits.
Horwood et al. (2018)	The following factor was associated with not breastfeeding at week 14: urban mothers, those with electricity.
Horwood et al. (2020)	Mothers in South Africa, raised hygiene concerns with expressed milk. This was coupled with the lack of infrastructure to support it, such as a fridge at home. Given the poor sanitation in low-income settlements where many women lived, they were concerned the expressed milk might become contaminated.
Hunter-Adams et al. (2016)	Participants mainly resided in shared accommodations like larger houses, apartments, or subdivided warehouses. Participants living in overcrowded and substandard housing conditions in Cape Town felt that these living conditions were stressful and contributed to their perception that breastfeeding was impractical.
Kyei et al. (2014)	Urban women may be working outside home so may not have enough time to breastfeed their babies for longer periods. This study indicates that the duration of breastfeeding in Vhembe has reduced.
Ndirangu et al. (2018)	NR

Smuts et al. (2008)	Toilet facilities (31 to 96%), tap water (9 to 38%), electricity (8 to 51%) varied among provinces. A large proportion of the study population did not have access to basic services. Differences were observed within and between provinces. Breast-feeding was similar in the two provinces up to the age of 18 months, but differed for 18- to 24-month-old children.
Stumbitz and Jaga (2020)	A lack of a safe environment, hindered access to public transport, and high levels, of stress from crime, gender-based violence and poverty has an impact on these women's capacity to pay attention to their lactating bodies.
Swarts et al. (2010)	The majority of the women in this study came from a poor socio-economic background and they would not be able to formula feed their infants safely and sustainably. Many of these infants of mothers who did not have access to water and flush toilets may then get diarrhea, infections and malnutrition that may even lead to death. Of the 72% of women who chose breastfeeding as first infant feeding method, only 42 (58%) were intending to breastfeed for the first six months 24 women breastfed for more than twelve months.

Abbreviations; NR: Not reported

Table S3.13: Orginin and migration, breastfeeding initiation, Western Europe

Included studies	*Impact of* ***origin and migration on breastfeeding initiation***
Bournez et al. (2017)	NR
Bonet et al. (2013)	NR
Brenne et al. (2018)	The duration of residence in Germany (among migrants) in years had no influence on the start of breastfeeding.
Camier et al. (2020)	Migrant motherhood: Positively related to patterns with higher BF rates.
Castebon et al. (2020)	NR
Frenoy et al. (2021)	Homeless families with diverse cultural backgrounds and migration histories from breastfeeding-normative countries displayed higher rates of breastfeeding. Mothers from Africa, both sub-Saharan and elsewhere, had higher rates of breastfeeding initiation than non-African mothers. Specifically, 90% of African-born mothers initiated breastfeeding compared to 80% of non-African mothers.
Girard et al. (2016)	Mothers born outside the cohort country (France) were more likely to breastfeed. Immigrant status increased the odds of breastfeeding initiation. Country of birth was a strong predictor breastfeeding initiation.

Guajardo-Villar et al. (2022)	Furthermore, The exclusive BF initiation rate for mothers living in departments where immigrants comprised 30% of the population was −11.8 [−25.6, −0.7] points lower than that for mothers living in departments with a 10% immigrant population (the average). The contextual variable most associated with mixed BF initiation rates was the proportion of immigrants in the department population. Rates were −3.8 [−10.1, 6.6] points lower for mothers from departments with a 30% immigrant population than for those living in departments with an average proportion of immigrants (10%). Furthermore, the exclusive BF initiation rate decreased more than the average in mothers living in departments where immigrants comprised 5% and 30% of the population by −10.0 [−11.9, −8.1] and −11.4 [−19.2, −2.9] points, respectively (vs. −9.6 points on average). Conversely, mixed BF initiation rates increased slightly more than the average in mothers who lived in a department with a 5% and 30% immigrant population (+5.9 [4.1, 7.9] and +6.5 [0.6, 18.2], respectively, vs. +5.1 points on average).
Lange et al. (2017)	German nationality, was negatively associated with exclusive breastfeeding intention.
Kersuzan et al. (2018)	Whereas only 67% of the mothers in the majority population breastfed their child at birth, 75% of the descendants of immigrants did so, and 88% of the immigrants. Breastfeeding at birth is more frequent among immigrants (92%) than among those from the European Union (87%), with those from sub-Saharan Africa (85%) positioned in the middle. Breastfeeding at birth is more common among natives with parents who were both born in the Maghreb or sub-Saharan Africa (82% and 92%, respectively), while descendants with only one parent from these countries and the other from another European Union country are no more likely to breastfeed than natives in the majority group. Among native parents, having an immigrant background appears only to have a minor impact on the likelihood of breastfeeding. Among immigrant mothers, their spouse’s origin has little effect on breastfeeding. Breastfeeding rates remain higher when the mother is an immigrant, regardless of the type of marriage. Having an immigrant husband therefore increases the likelihood that a mother born in France will breastfeed. Due to the endogamy of the marriages, immigrants and

	descendants of immigrants frequently have spouses of the same origin which has a positive effect on breastfeeding at birth.
Robert et al. (2014a)	NR
Wagner et al. (2019)	Foreign-born mothers had a higher breastfeeding initiation rate than native French mothers. But, this effect was observed for only any breastfeeding: mother's country of birth had no effect on predominant breastfeeding.

Abbreviations; BF: Breastfeeding, NR: Not reported

Tabel 3.14: Origin and migration, breastfeeding initiation, Southern Africa

Included studies	*Impact of* ***origin and migration on breastfeeding initiation***
Hunter-Adams et al. (2016)	Participants' immigration affected their ability to breastfeed, due to the impact of living in an new and unknown environment.

Table S3.15: Origin and migration, breastfeeding duration, Western Europe

Included studies	*Impact **of origin and migration on breastfeeding duration***
Bournez et al. (2017)	Couples where neither parent was born in France introduced complementary feeding (CF) before 4 months more often than couples with both parents born in France. When the mother was foreign-born but the father was French-born, CF introduction after 6 months was less common than in families with both parents born in France. Although foreign-born mothers breastfed for longer durations, they were more likely to start CF before 4 months and less likely after 6 months compared to French-born mothers.
Bonet et al. (2013)	Breastfeeding rates at 4 months of infant's age (both any and almost exclusive breastfeeding rates) were higher among those with a foreign family origin.
Brenne et al. (2018)	More acculturated women show a greater risk of weaning within the first six months post-partum than less acculturated women with a migration background. 6884 women were contacted for the T2 interviews in the postnatal wards, 90.4% (n = 6220) of these had started breastfeeding: 93% of second- and third-generation migrant women and 88.2% of non-migrants. Of 747 women who had agreed to a phone survey six months after delivery (T3), 605 could be contacted (response 81%). At this time, 312 (51.6%) of the 605 women in this subgroup were still breastfeeding.
Camier et al. (2020)	Migrant motherhood: Positively related to patterns with very long BF duration but negatively related to patterns with 'later CF and longer BF'.
Castebon et al. (2020)	Characteristics like birthplace were associated with breastfeeding (BF) duration. Foreign-born women were more likely to combine BF and work, potentially serving as a model for native-born mothers.
Frenoy et al. (2021)	70% of sub-Saharan and 56% of other African-born mothers breastfed for at least 6 months, while only 50% of non-African mothers did. Factors like the child's birth before migration were linked to longer breastfeeding, while migrating due to violence was associated with shorter breastfeeding durations compared to other migration reasons.

Girard et al. (2016)	NR
Guajardo-Villar et al. (2022)	NR
Lange et al. (2017)	NR
Kersuzan et al. (2018)	Six months: 45% of immigrants breastfeed versus 20% of natives. Immigrant mothers also breastfeed for longer than natives, especially those from sub-Saharan Africa, 55% of whom breastfed until the infant was six months old, versus around 40% of immigrants from all other countries. Mothers with two parents born in a sub-Saharan African country breastfed more at six months (40%).
Robert et al. (2014a)	Mothers of foreign origin were less likely to stop any BF and exclusive BF than other mothers. Exclusive and any breastfeeding durations were independently positively associated ($P < 0.05$) with foreign-born mothers.
Wagner et al. (2019)	Foreign-born mothers had longer breastfeeding duration than native French mothers. But, this effect was observed for only any breastfeeding: mother's country of birth had no effect on predominant breastfeeding.

Abbreviations; BF: Breastfeeding, CF: Complementary Feeding, NR: Not Reported

Table S3.16: Ethnicity and cultural aspects, breastfeeding initiation, Western Europe

Included studies	*Impact of* ***ethnicity and cultural aspects on breastfeeding initiation***
Bernard et al. (2016)	The results suggest that women living in a country or region where Catholicism has historically dominated are less likely to initiate breast feeding, and that breast feeding promotion policies should be adapted to better fit populations' cultural and religious norms. The study found a negative correlation (r=−0.30) between the proportion of Catholics and the rate of breast feeding initiation in Western countries. This correlation was consistent when using within-country data in France (r=−0.27). These correlations remained strongly negative after controlling for GDP per capita and population density. On the other hand, the study found a positive correlation between breast feeding initiation rate and the proportion of Protestants (r=0,31). The proportions of 'no religion' was weak and negative correlated with breast feeding initiation rates in France (r=-0,07). Countries in which the breast feeding initiation rate was below 80% are historically Catholic (eg, France). In conclusion, the analysis suggests a new population-level factor influencing breast feeding initiation across and within Western countries: the proportion of Catholics.
Frenoy et al. (2021)	Homeless families with diverse cultural backgrounds and migration histories from breastfeeding-normative countries displayed higher rates of breastfeeding.
Girard et al. (2016)	Regional or cultural differences in breastfeeding behaviours are likely to persist.
Quittner et al. (2017)	The Turkish group had the highest rate of complete breastfeeding and the Antillean/ Aruban and Surinamese groups had the lowest. Amsterdam infants of Turkish origin were most often breastfed, infants of Antillean/Aruban or Surinamese origin the least often. This was true for all ages. Overall conclusion: Breastfeeding rates differ for ethnic origin groups and changes over time differ possibly by group as well.

Van Rossem et al. (2009b)	Relative to native Dutch mothers, starting breastfeeding was significantly higher in all non-native groups. Adjustment for educational level strengthened the associations. The higher percentage of people from ethnic minorities who start breastfeeding seems to have a cultural basis: The Koran recommends 2 years of breastfeeding, which may underlie the high starting rates of Mediterranean women in our study. Overall conclusion: This study shows that more non-native mothers start breastfeeding. Overall, 90.6% of women started breastfeeding after delivery. Lowest percentage among the Dutch native (89.1%) and highest among the Mediterranean second-generation women (98.6%; $p<0.001$). Mediterranean-Turkish mothers have high breastfeeding rates.
Wagner et al. (2019)	Foreign-born mothers had higher initiation rates than native French mothers, influenced by cultural values and practices from their origin countries.

Abbreviations; GDP: Gross Domestic Product

Table S3.17: Ethnicity and cultural aspects, breastfeeding initation, Southern Africa

Included studies	*Impact of* ***ethnicity and cultural aspects on breastfeeding initiation***
Seabela et al. (2023)	Mothers in this study reported traditional practices that affect their efforts to breastfeed, even exclusively breastfeeding their infants. In most African societies, performing traditional rituals on babies to protect them from evil spirits is a norm. Mothers shared some family beliefs and practices but they were still divided between societal and cultural norms and traditional beliefs as facilitators or barriers to breastfeeding.

Tabel S3.18: Ethnicity and cultural aspects, breastfeeding duration, Western Europe

Included studies	*Impact of* ***ethnicity and cultural aspects on breastfeeding duration***
Bernard et al. (2016)	NR
Frenoy et al. (2021)	NR
Girard et al. (2016)	Regional or cultural differences in breastfeeding behaviours are likely to persist.
Quittner et al. (2017)	NR
Van Rossem et al. (2009b)	Generally, although more mothers from ethnic minorities in The Netherlands start breastfeeding than native mothers do, they are less likely to continue.
Wagner et al. (2019)	Foreign-born mothers had longer duration than native French mothers, influenced by cultural values and practices from their origin countries.

Abbreviations; NR: Not Reported

Table S3.19: Access to affordable health services of decent quality, breastfeeding, Southern Africa

Included studies	*Impact of* ***access to affordable health services of decent quality on breastfeeding***
Horwood et al. (2019)	Access to health services for this population is good and comparable to the general population in South Africa.

Table S3.20: Food insecurity, breastfeeding, Southern Africa

Included studies	*Impact of **food insecurity on breastfeeding***
Horwood et al. (2019)	Mothers in KwaZulu-Natal, South Africa reported significant food insecurity for themselves and for their children. Among all participating mothers, 61.9% of the mothers reported that in the past 4 weeks she had to miss a meal because of lack of resources to buy food, and 15.8% of the mothers reported this had happened frequently (more than 10 times in 4 weeks). Food insecurity was similar among informal traders and domestic workers.
Hunter-Adams et al. (2016)	Cross-border migrants in Cape Town identified low rates of breastfeeding as being influenced by poor diets. The cost of formula feeding was perceived as a significant barrier to breastfeeding back in participants' home countries.
Mphasha et al. (2023)	69.8% of caregivers in Limpopo Province, South Africa did not give infant formula due to cost.

File S4. PRISMA 2020 Checklist

Section and Topic	Item #	Checklist item	Location where item is reported
**TITLE**			
Title	1	Identify the report as a systematic review.	OK
**ABSTRACT**			
Abstract	2	See the PRISMA 2020 for Abstracts checklist.	OK
**INTRODUCTION**			
Rationale	3	Describe the rationale for the review in the context of existing knowledge.	OK
Objectives	4	Provide an explicit statement of the objective(s) or question(s) the review addresses.	OK
**METHODS**			
Eligibility criteria	5	Specify the inclusion and exclusion criteria for the review and how studies were grouped for the syntheses.	OK
Information sources	6	Specify all databases, registers, websites, organisations, reference lists and other sources searched or consulted to identify studies. Specify the date when each source was last searched or consulted.	OK
Search strategy	7	Present the full search strategies for all databases, registers and websites, including any filters and limits used.	OK
Selection process	8	Specify the methods used to decide whether a study met the inclusion criteria of the review, including how many reviewers screened each record and each report retrieved, whether they worked independently, and if applicable, details of automation tools used in the process.	OK
Data collection process	9	Specify the methods used to collect data from reports, including how many reviewers collected data from each report, whether they worked independently, any processes for obtaining or confirming data from study investigators, and if applicable, details of automation tools used in the process.	OK
Data items	10a	List and define all outcomes for which data were sought. Specify whether all results that were compatible with each outcome domain in each study were sought (e.g. for all measures, time points, analyses), and if not, the methods used to decide which results to collect.	OK
	10b	List and define all other variables for which data were sought (e.g. participant and intervention characteristics, funding sources). Describe any assumptions made about any missing or unclear information.	NA
Study risk of bias assessment	11	Specify the methods used to assess risk of bias in the included studies, including details of the tool(s) used, how many reviewers assessed each study and whether they worked independently, and if applicable, details of automation tools used in the process.	OK
Effect measures	12	Specify for each outcome the effect measure(s) (e.g. risk ratio, mean difference) used in the synthesis or presentation of results.	NA
Synthesis methods	13a	Describe the processes used to decide which studies were eligible for each synthesis (e.g. tabulating the study intervention characteristics and comparing against the planned groups for each synthesis (item #5)).	OK
	13b	Describe any methods required to prepare the data for presentation or synthesis, such as handling of missing summary statistics, or data conversions.	OK
	13c	Describe any methods used to tabulate or visually display results of individual studies and syntheses.	OK
	13d	Describe any methods used to synthesize results and provide a rationale for the choice(s). If meta-analysis was performed, describe the model(s), method(s) to identify the presence and extent of statistical heterogeneity, and software package(s) used.	NA
	13e	Describe any methods used to explore possible causes of heterogeneity among study results (e.g. subgroup analysis, meta-regression).	NA
	13f	Describe any sensitivity analyses conducted to assess robustness of the synthesized results.	NA
Reporting bias assessment	14	Describe any methods used to assess risk of bias due to missing results in a synthesis (arising from reporting biases).	NA
Certainty assessment	15	Describe any methods used to assess certainty (or confidence) in the body of evidence for an outcome.	NA

4. PRISMA 2020 Checklist

Section and Topic	Item #	Checklist item	Location where item is reported
**RESULTS**			
Study selection	16a	Describe the results of the search and selection process, from the number of records identified in the search to the number of studies included in the review, ideally using a flow diagram.	OK
	16b	Cite studies that might appear to meet the inclusion criteria, but which were excluded, and explain why they were excluded.	OK
Study characteristics	17	Cite each included study and present its characteristics.	OK (Suppl)
Risk of bias in studies	18	Present assessments of risk of bias for each included study.	OK (Suppl)
Results of individual studies	19	For all outcomes, present, for each study: (a) summary statistics for each group (where appropriate) and (b) an effect estimate and its precision (e.g. confidence/credible interval), ideally using structured tables or plots.	OK
Results of syntheses	20a	For each synthesis, briefly summarise the characteristics and risk of bias among contributing studies.	OK
	20b	Present results of all statistical syntheses conducted. If meta-analysis was done, present for each the summary estimate and its precision (e.g. confidence/credible interval) and measures of statistical heterogeneity. If comparing groups, describe the direction of the effect.	NA
	20c	Present results of all investigations of possible causes of heterogeneity among study results.	NA
	20d	Present results of all sensitivity analyses conducted to assess the robustness of the synthesized results.	NA
Reporting biases	21	Present assessments of risk of bias due to missing results (arising from reporting biases) for each synthesis assessed.	NA
Certainty of evidence	22	Present assessments of certainty (or confidence) in the body of evidence for each outcome assessed.	NA
**DISCUSSION**			
Discussion	23a	Provide a general interpretation of the results in the context of other evidence.	OK
	23b	Discuss any limitations of the evidence included in the review.	OK
	23c	Discuss any limitations of the review processes used.	OK
	23d	Discuss implications of the results for practice, policy, and future research.	OK
**OTHER INFORMATION**			
Registration and protocol	24a	Provide registration information for the review, including register name and registration number, or state that the review was not registered.	OK
	24b	Indicate where the review protocol can be accessed, or state that a protocol was not prepared.	OK
	24c	Describe and explain any amendments to information provided at registration or in the protocol.	OK
Support	25	Describe sources of financial or non-financial support for the review, and the role of the funders or sponsors in the review.	OK
Competing interests	26	Declare any competing interests of review authors.	OK
Availability of data, code and other materials	27	Report which of the following are publicly available and where they can be found: template data collection forms; data extracted from included studies; data used for all analyses; analytic code; any other materials used in the review.	OK

From: Page MJ, McKenzie JE, Bossuyt PM, Boutron I, Hoffmann TC, Mulrow CD, et al. The PRISMA 2020 statement: an updated guideline for reporting systematic reviews. BMJ 2021;372:n71. doi: 10.1136/bmj.n71.
This work is licensed under CC BY 4.0. To view a copy of this license, visit https://creativecommons.org/licenses/by/4.0/

File S5. PRISMA 2020 for Abstracts Checklist

Section and Topic	Item #	Checklist item	Reported (Yes/No)
**TITLE**			
Title	1	Identify the report as a systematic review.	Yes
**BACKGROUND**			
Objectives	2	Provide an explicit statement of the main objective(s) or question(s) the review addresses.	Yes
**METHODS**			
Eligibility criteria	3	Specify the inclusion and exclusion criteria for the review.	No
Information sources	4	Specify the information sources (e.g. databases, registers) used to identify studies and the date when each was last searched.	Yes
Risk of bias	5	Specify the methods used to assess risk of bias in the included studies.	Yes
Synthesis of results	6	Specify the methods used to present and synthesise results.	No
**RESULTS**			
Included studies	7	Give the total number of included studies and participants and summarise relevant characteristics of studies.	Yes
Synthesis of results	8	Present results for main outcomes, preferably indicating the number of included studies and participants for each. If meta-analysis was done, report the summary estimate and confidence/credible interval. If comparing groups, indicate the direction of the effect (i.e. which group is favoured).	Yes
**DISCUSSION**			
Limitations of evidence	9	Provide a brief summary of the limitations of the evidence included in the review (e.g. study risk of bias, inconsistency and imprecision).	No
Interpretation	10	Provide a general interpretation of the results and important implications.	Yes
**OTHER**			
Funding	11	Specify the primary source of funding for the review.	No
Registration	12	Provide the register name and registration number.	Yes

From: Page MJ, McKenzie JE, Bossuyt PM, Boutron I, Hoffmann TC, Mulrow CD, et al. The PRISMA 2020 statement: an updated guideline for reporting systematic reviews. BMJ 2021;372:n71. doi: 10.1136/bmj.n71. This work is licensed under CC BY 4.0. To view a copy of this license, visit https://creativecommons.org/licenses/by/4.0/
